# Towards Better Understanding of Pea Seed Dormancy Using Laser Desorption/Ionization Mass Spectrometry

**DOI:** 10.3390/ijms18102196

**Published:** 2017-10-21

**Authors:** Monika Cechová, Markéta Válková, Iveta Hradilová, Anna Janská, Aleš Soukup, Petr Smýkal, Petr Bednář

**Affiliations:** 1 Regional Centre of Advanced Technologies and Materials, Department of Analytical Chemistry, Faculty of Science, Palacký University, 17. Listopadu 12, 771 46 Olomouc, Czech Republic; monicechova@gmail.com (M.C.); ponizilova.marketa@seznam.cz (M.V.); 2 Department of Botany, Faculty of Science, Palacký University, Šlechtitelů 27, 783 71 Olomouc, Czech Republic; HradilovaI@seznam.cz (I.H.); petr.smykal@upol.cz (P.S.); 3 Department of Experimental Plant Biology, Faculty of Science, Charles University, Viničná 5, 128 44 Prague, Czech Republic; janska@natur.cuni.cz (A.J.); asoukup@natur.cuni.cz (A.S.)

**Keywords:** pea, fatty acid, seed coat, seed dormancy, seed hardness, laser desorption-ionization mass spectrometry, imaging mass spectrometry, multivariate statistics

## Abstract

Seed coats of six pea genotypes contrasting in dormancy were studied by laser desorption/ionization mass spectrometry (LDI-MS). Multivariate statistical analysis discriminated dormant and non-dormant seeds in mature dry state. Separation between dormant and non-dormant types was observed despite important markers of particular dormant genotypes differ from each other. Normalized signals of long-chain hydroxylated fatty acids (HLFA) in dormant JI64 genotype seed coats were significantly higher than in other genotypes. These compounds seem to be important markers likely influencing JI64 seed imbibition and germination. HLFA importance was supported by study of recombinant inbred lines (JI64xJI92) contrasting in dormancy but similar in other seed properties. Furthemore HLFA distribution in seed coat was studied by mass spectrometry imaging. HLFA contents in strophiole and hilum are significantly lower compared to other parts indicating their role in water uptake. Results from LDI-MS experiments are useful in understanding (physical) dormancy (first phases of germination) mechanism and properties related to food processing technologies (e.g., seed treatment by cooking).

## 1. Introduction

Seed germination is a key step of plant life predetermining spread of particular plant species on the Earth [1]. In a population of wild seeds only a certain fraction of individuals starts to germinate in favorable conditions. Their inactive counterparts can survive adverse periods and thus ensure continuation of given plant species for long time. The extent of seed inactivity is expressed by dormancy [2]. Dormancy is a regulatory and adaptive trait in virtually all seed-plant species. There are several types of seed dormancy, with one of them being physical dormancy, executed by water impermeable seed coat [3]. This type is prevalent in legumes [4]. Its detailed description and classification, importance for agriculture and food industry and connection with other seed properties (i.e., seed hardness, pre-harvest sprouting, etc.) is given in two our recent papers [4,5] and in citations listed therein.

Activation of a seed starts with water imbibition and penetration of gases through the seed coat (their restriction is described as physical dormancy) [3]. These processes are connected with structure and chemical composition of the most outer cell layers of seed coats [4]. A number of analytical techniques was used for chemical analysis of seed composition (including characterization of separated seed coat) starting with a selective staining [4], over standard instrumental techniques, i.e., gas chromatography with flame ionization detection (GC-FID) [6], high performance liquid chromatography (HPLC) with ultraviolet/visible (UV/Vis) spectrophotometric detection [7,8] up to hyphenated techniques, first of all gas chromatography-mass spectrometry (GC/MS) and liquid chromatography mass spectrometry (LC/MS) [9,10,11]. All these techniques utilize an interaction of plant material with a solvent (soaking or liquid extraction), often in combination with further chemical treatment (i.e., hydrolysis and derivatization). GC/MS was used for analysis of saponified and methylated seed coats [12]. These techniques dispose with high selectivity towards extractable analytes.

Number of techniques allow direct analysis of seeds in dry state or after a very short contact with a solvent (i.e., spray covering of surface with a matrix prior to a matrix assisted laser desorption/ionization mass spectrometric analysis (MALDI-MS), etc.) preventing (or significantly restricting) the initiation of the imbibition processes during sample preparation or (reversely) to study the changes after defined wetting of seed coat. Especially, spectrometric techniques such as MALDI-MS, desorption electrospray mass spectrometry (DESI-MS), direct analysis in real time mass spectrometry (DART-MS), nuclear magnetic resonance spectroscopy (NMR), Fourier-transform infrared spectroscopy (FTIR) and laser ablation–inductively coupled plasma–mass spectrometry (LA-ICP-MS) have been already used for analysis of seeds as recently reviewed [13,14,15,16,17,18,19,20]. Notably none of those techniques was used for direct measurement of separated seed coat tissue from embryo and endosperm.

Majority of later methods possesses spatial information (imaging methods). Perhaps the most widespread and powerful technique for the analysis of seed surface layers with respect to organic molecules is laser desorption/ionization imaging mass spectrometry. It provides high versatility often with acceptable selectivity and sensitivity. Mass spectrometry is very popular technique in proteomic and metabolic profiling of plant tissues. MALDI-MS technique combined with gel electrophoresis was used in study of proteomic composition of *Medicago truncatula* [21], *Lotus japonicas* and other legume seeds [22]. MALDI-mass spectrometry imaging (MALDI-MSI) protocols for the detection of small molecules in cryodissected immature barley grains are described in the work of Peukert et al. [23]. Bhandari et al. published a comprehensive high resolution MS imaging analysis of cryosections of two types of seeds (oil-seed rape and wheat) and other plant tissues (wheat rachis, stem base, rice root) concerning with germination and seed maturation [24]. Besides, Gorzolka et al. have shown the possibility of MALDI-MS imaging for spatial-temporal metabolite profiling during the germination of barley seeds [25].

To the best of our knowledge this is the first report of the utilization of laser desorption/ionization mass spectrometry (LDI-MS) for the analysis of seed coat composition in the relation to the level of seed dormancy. The main aim of this work was to study the potential of laser desorption/ionization mass spectrometry for the surface analysis of pea seed coat in mature dry state with respect to physical dormancy. Multivariate statistics on raw MS data was used for classification of pea genotypes and lines and obtained outputs correlated with level of dormancy. Dormant and non-dormant genotypes (possessing different propensity to imbibition and germination) were profiled and content of hydroxylated long-chain fatty acids (HLFA) was identified as the important discriminating factor. The obtained information is significant also for agricultural and food industry.

## 2. Results and Discussion

### 2.1. LDI-MS Measurement and Utilization of PCA and OPLS-DA for Data Analysis

**Figure 1:**
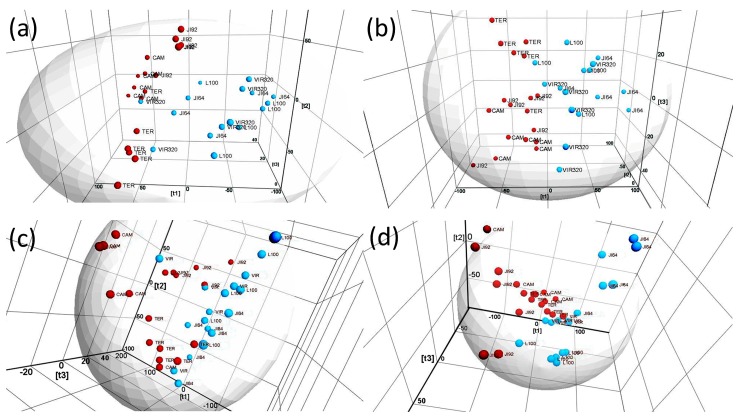
3D Score plots obtained by Principal Component Analysis of MALDI-MS and LDI-MS data. Upper plots—measurement in negative ionization mode, (**a**) with matrix and (**b**) without matrix; bottom plots—measurement in positive ionization mode, (**c**) with matrix and (**d**) without matrix; dormant species are marked with blue and non-dormant ones with red bullets; Pareto scaling and marker intensity threshold 1000 was used for data matrix processing.

LDI-MS spectra of outer surface of six different genotypes (JI92, Cameor, Terno, JI64, VIR320 and L100) were measured in positive and negative ion mode. These genotypes represent both wild and domesticated pea types commonly used in genetic and biological studies [5]. They contain both pigmented and non-pigmented seed coats. Strong signals at *m*/*z* 98.9769, 112.9229, 154.9325, 196.9439, 206.9943, 291.1685, 317.11198, 369.1229, 377.1309 and 485.3076 were observed in positive mode and those at *m*/*z* 121.0060, 132.0010, 143.9992, 156.0039, 169.0079, 193.0115, 204.0037, 214.0169, 217.0682, 268.0770, 282.1081 306.1075, 319.1063 and 323.1524 in negative ion mode. Differences in those major signals, however, do not allow a resolution of wild/dormant from cultivated/non-dormant genotypes. Although certain differences among some genotypes are visible (i.e., Terno in positive and Cameor in negative ion modes), the direct interpretation of MS spectra do not allow to find characteristic signals for cultivation/dormancy (raw LDI-MS and MALDI-MS spectra are given in supplemental material, Figure S1a–d). Since comprehensive evaluation of differences by direct raw MS spectra comparison was not possible, multivariate analysis was applied. Principal Component Analysis (PCA) and Orthogonal Partial Least Squares Discriminant Analysis (OPLS-DA) are generally the most proven methods to differentiate between classes in highly complex data sets. We started with application of classical PCA to visualize the chemical differences among samples by unsupervised (independent) way. Utilization of PCA as starting point during multidimensional data treatment was recommended for instance by Worley and Powers for metabolomics representing similar exploratory area [26]. Figure 1 shows the 3D Score plots obtained by PCA of raw LDI-MS and MALDI-MS data in positive and negative ion modes.

Despite a relatively large variation of replicated measurements, separation of dormant and non-dormant genotypes is evident in each ionization mode regardless whether matrix is used or not. Roughly, non-dormant samples are located on left and dormant ones on the right side in each 3D Score plot, respectively. These results suggest that laser desorption ionization mass spectrometry can be used for classification (resolution) of pea genotypes with regard to surface composition related to domestication/dormancy by direct analysis of dry maturated seeds as alternative method to classical study of germination. It should be emphasized that the resolution can be achieved without selection and knowledge of the origin of markers (signals significantly differing in content between dormant and non-dormant samples). Nevertheless, identification of one important group of markers was successful as described in next Chapters. In negative ion mode (Figure 1a,b), the first three dimensions of the Score plots explain 78.53% and 76.46% of data variability in matrix assisted and matrix free experiments, respectively. In positive ion mode similar level of explained variability was achieved (80.28% and 75.58%, respectively). Complete datasets related to those Score Plots are given in supplementary material, Table S1a–d. Surface morphology differs significantly among genotypes (e.g., presence of “gritty” surface on seed coats of some genotypes, Figure S2). Such differences at surface can alter the mechanism of water penetration through the seed coat (the mechanism of seed imbibition and consequently dormancy)—from a biological point of view—as well as ionization efficiency during LDI-MS measurement—considering the used analytical methodology. To conclude, the resolution of dormant and non-dormant species was unambiguously achieved but surface morphology affects significantly the distribution of particular data-points.

As already mentioned, the resolution of dormant and non-dormant genotypes is achieved regardless the presence of matrix. Application of matrix using standard automatized solution spraying technique causes torsion of samples attached to MALDI plate. We observed that this process is more pronounced in non-dormant genotypes. Such torsion is connected with a tension among different seed coat cell layers during their contact with water and its extent is possibly due to thiner seed coat of non-dormant compared to dormant species [5]. When occurring on an intact seed in nature, this phenomenon could contribute to seed coat rupture, opening the seed for water and (consequently) accelerating germination [5]—hence the stronger connection of the torsion on MALDI plate with non-dormant (faster germinating) seed coats. Although the measurement of deformed seed coat pieces was possible and similar degree of distinction of samples according to dormancy level was achieved in matrix assisted and matrix free experiments, the changes of the surface cell layers by their swelling with the applied solvent during and after spraying represent a phenomenon negatively affecting both the sample preparation as well as signals in MS spectra. The matrix-free method should be therefore further preferred from methodological point of view. The effect of matrix presence on the significance of identified signals in terms of OPLS-DA (the position of corresponding markers in particular S-plots) will be discussed later.

The differences in chemical composition of individual dormant genotypes were studied against non-dormant genotypes (all three non-dormant genotypes as reference set) by OPLS-DA. Obtained signals almost completely differ when compared particular dormant genotypes with two exceptions (given by italics, Table S2) that could contribute to differences in the process of water imbibition. Signals of particular hydroxylated long-chain fatty acids appeared among the most important markers of JI64 genotype as discussed later in more details.

**Figure 2:**
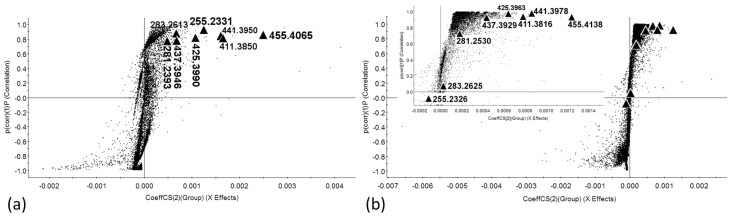
S-plots obtained by OPLS-DA of JI64 a JI92 genotypes. (**a**) Matrix free experiments; (**b**) matrix assisted experiments (inset in (**b**) shows zoomed dormant part of the appropriate S-plot); enlarged triangles indicate identified markers of physical dormancy.

As already indicated, the distinctness in seed characters (i.e., size, pigmentation, surface properties, etc.) influences the LDI-MS experiment. We therefore further focused on JI64 (D) and JI92 (N) pair that is less contrasting in term of seed coat appearance but strongly different in seed coat permeability and dormancy (for details see Section 3.2. and [5]). Note that those two genotypes are very well separated by unsupervised PCA (see all 3D Score Plots, Figure 1). Data obtained by their measurement were also analyzed by OPLS-DA. Figure 2 shows the S-plots reflecting the differences in signals between both genotypes in negative ion mode (all markers and their coordinates in S-plots are given for both ionization modes in supplemental material, Table S3a–d).

The area of significant signals (further referred as dormancy markers, DM) in each S-plot corresponds with low risk region [27] that is given in this study as a box with following coordinates: CoeffCS(2) = 10–100% and p(corr)(1) = 75–100% from the highest value at CoeffCS(2)-axis (*x*-axis) and p(corr)(1)-axis (*y*-axis), respectively, in “dormant” part (the first quadrant) of the S-plot. Signals at *m*/*z* 255.2331, 281.2393, 283.2613, 411.3850, 425.3990, 437.3946, 441.3950 and 455.4065 fell into this region and are thus classified as significant DM in matrix free LDI-MS experiments (Figure 2a). Analogous signals were observed in MALDI-MS spectra as well but some of them out of low risk region of related S-plot (Figure 2b).

Those signals correspond well with theoretical *m*/*z* values of deprotonated molecules of common fatty acids (palmitic, oleic and stearic acid) and, perhaps more importantly, with hydroxylated long chain fatty acids (HLFA). Table 1 shows details of these markers. HLFA have been already considered as hydrophobic components increasing impermeability of cutin layers for water [5,6].

There are several former reports confirming that LDI-MS is generally suitable technique for analysis of fatty acids. Pirkl et al. dealt with the direct profiling of fatty acids in insect tissues by LDI-MS [28], for instance. Budimir et al. published laser desorption/ionization on porous silicon mass spectrometric analysis of nonadecanoic acid and heneicosanoic acid [29]. Besides, Shroff et al. have shown that MALDI-MS can be used for very sensitive analysis of various acids including fatty acids in negative ion mode [30]. Since the defined standards of HLFA are not commercially available, our LDI-MS method was verified using a mixture of common palmitic and oleic acid. Both acids provided the signal of appropriate anions when measured separately and also when applied at the pea seed coat surface, i.e., signals at *m*/*z* 255.2312 and 281.2465 were achieved in both experiments (deviation from theoretical mass, dtm, was 6.7 and 7.5 ppm, respectively, corresponding MS spectra are given in supplementary material, Figure S3a,b). Those results confirm the capability of our method to analyze fatty acids present on the surface of seed coats. Tandem MS/MS experiments confirmed the identity of HLFA markers on seed coat surface (see Figure S4 showing LDI-MS/MS spectrum of dihydroxyoctacosanoate measured with Trap CE 30 eV yielding the highest signal of characteristic fragments). Loss of water from parent ion was observed providing the fragment at *m*/*z* 437.3940 (C_28_H_53_O_3_^−^). This loss suggests the presence of a hydroxyl group in the molecule. The signal of fragment arising by two consequent losses of water (confirming the presence of two hydroxyls in parent ion) was also observed but with low intensity (fragment at *m*/*z* 419.3841, C_28_H_51_O_2_^−^). Loss of CO_2_ was not observed. Structure of dihydroxyoctacosanote fragments at *m*/*z* 183.1372, 253.2503 and 267.2664 was considered as well. The fragment at *m*/*z* 183.1372 corresponds with mass of undecenoate that can be formed by a cleavage of eleven-carbon chain from carboxyl end of dihydroxyoctacosanoate and elimination of water (C_11_H_19_O_2_^−^). The latter two fragments could arise by a cleavage of C17 and C18 chain and elimination of water (C_17_H_33_O^−^ and C_18_H_35_O^−^, respectively). A higher deviation of measured *m*/*z* values from theoretical ones is due to weak signal of rising fragments in MS/MS spectra. The fragmentation pattern suggests the C10 and/or C12 hydroxylation, however, the detailed elucidation of the position of hydroxyl is the objective of future research. Analogous fragmentation pattern was described by Nilsson et al. [31]. Besides, the formation of fragments that do not bear a carboxy group was described also by Kerwin et al. [32]. The presence of the above discussed fragments further confirms the identity of the found HLFA. The other HLFA did not provide utilizable signals of fragments in MS/MS spectra due to lower intensity of corresponding parent ions. Loss of water (but with very low intensity) was observed also in MS/MS spectra of monohydroxylated HLFA (i.e., hydroxyhexacosanoate, parent ion at *m*/*z* 411.3865 and hydroxyheptacosanoate, parent ion at *m*/*z* 425.3927).

**Figure 3:**
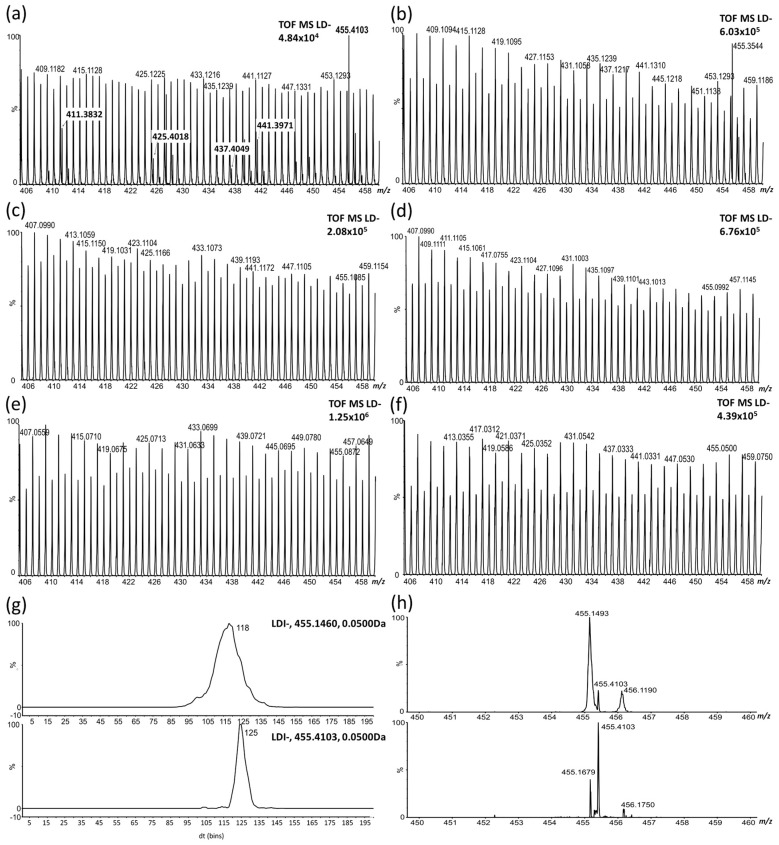
LDI-MS spectra in negative ionization mode. (**a**) external seed coat surface of JI64 genotype; (**b**) external seed coat surface of JI92; (**c**) internal surface of JI 64; (**d**) internal surface of JI92; (**e**) seed coat cross section of JI64 (MSI); (**f**) seed coat cross section of JI92 (MSI); (**g**) upper mobilogram (record of ion mobility scan) reconstructed at *m*/*z* 455.1460 and bottom mobilogram at *m*/*z* 455.4103; (**h**) upper MS/MS spectrum averaged over the whole mobility range and bottom MS/MS spectrum averaged over the second mobility peak (with maximum at 125 bins).

The identified HLFA provided significantly higher signal in spectra of dormant JI64 genotype compared to non-dormant JI92 confirming the results of untargeted multivariate statistics. Figure 3 shows the MS spectra of external and internal surface of seed coat of both in dormancy contrasting genotypes.

Signals of HLFA are visible in the spectrum of external surface of JI64 (Figure 3a). They appear very close to another (stronger) signals with the same nominal mass (arising probably by a thermal destruction of polysaccharides and lignin). These HLFA signals are missing in the other LDI-MS spectra measured on external surface of JI92 genotype as well as on internal seed coat surface and its cross sections of both genotypes (Figure 3b–f). The resolution of the HLFA signals can be improved using ion mobility separation. The signal of dihydroxyoctacosanoate dominates in the spectrum obtained by averaging over its ion mobility peak and baseline correction being well separated from the interfering signal at *m*/*z* 455.1493 (Figure 3g shows mobilograms reconstructed for the interfering signal, upper trace and dihydroxyoctacosanoate, bottom trace; Figure 3h shows the spectrum averaged over the whole mobility range, upper spectrum and over the mobility peak with apex at 125 bins, bottom spectrum). Similar ion mobility separation can be achieved for the other HLFA.

The yield of signal of the identified HLFA with respect to sum of all signals present in spectra (normalized signal) was further optimized. The effect of ion source parameters on the normalized response of analytes and on the total ion current in MS spectra was studied in JI64 genotype. The effect of hexapole RF amplitude, sample plate voltage, laser energy and laser step rate on the normalized signal of dihydroxyoctacosanoate as the HLFA with the highest mass and response was studied (supplementary material, Figure S5). The effect of hexapole RF amplitude is quite significant. This parameter is important for focusing and transmission of ions in a lower vacuum region and it is *m*/*z* dependent in a broad range. The optimal value of this parameter is 300 V. Besides, the decreasing of sample plate voltage from the default 25 to 10 V caused utilizable improvement of signal. The optimal value of laser energy was set at 300 (dimensionless parameter, the value affects position of neutral density filter that attenuates/amplifies the laser beam to provide energy setting functionality). Laser step rate (velocity of the laser movement) influences the signal of dihydroxyoctacosanoate significantly as well (optimal value is 30). Slower motion of laser burns seed coats and HLFA signals are suppressed probably due to pyrolysis products that rise in more amounts. The effect of this parameter is connected with applied the energy of laser beam. However, for MSI mode (discussed in detail in the next chapter) operating with more focused laser beam to achieve better spatial resolution (beam size 60 µm, pixel size 50 × 50 µm) the optimal laser energy was lower (250–300). Higher value than 300 rapidly burnt analyzed seed coats.

### 2.2. Study of the Distribution of Hydroxylated Long-Chain Fatty Acids on the Pea Seed Coat Surface

**Figure 4:**
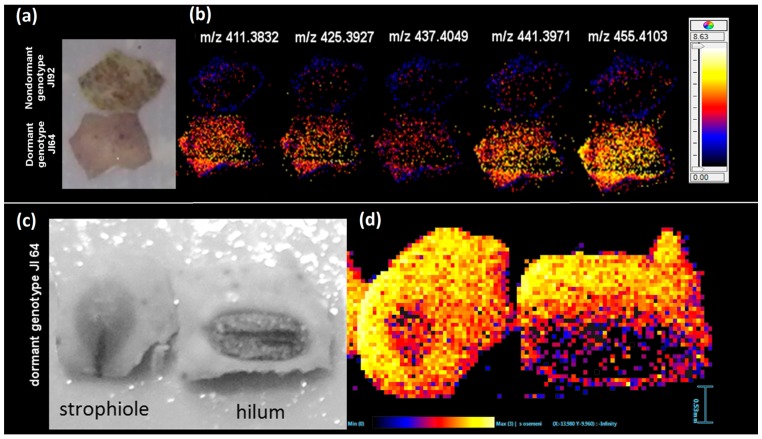
LDI-MSI analysis of external seed coat surfaces. (**a**) Optical image of analyzed seed coats used for mass imaging; (**b**) comparison of distribution of signals (*m*/*z* values) of hydroxylated fatty acids on seed coat of dormant JI64 (bottom mass images) and non-dormant JI92 (upper mass images); (**c**) optical image of parts of JI64 seed coats containing strophiole and hilum used for mass imaging; (**d**) distribution of dihydroxyoctacosanoate (*m*/*z* 455.4103) over the external surface containing strophiola and hilum; matrix free MSI measurement. Colors in the mass images, i.e., (**b**,**d**), correspond with intensity of particular HLFA markers (their amounts on seed coat surface) in MS spectra (yellow reflects the highest signal and black the lowest one as displayed by inserted color scales).

As already mentioned, water penetration through the seed coat is to certain extent driven by the composition of the outermost seed coat layer formed by hydrophobic compounds like cutin [4] and most likely also subcuticular lipids present in the macrosclereids. Low content of HLFA in some parts of seed coat outer surface (inhomogeneity of HLFA distribution over the outer surface) would be a primary place for water intrusion even when other parts of the surface are very homogeneous and rich in fatty acids. Imaging mass spectrometry was used to study the distribution of the HLFA previously detected as the dormancy markers as discussed in the previous chapter. Figure 4b compares the HLFA distribution (signals of particular *m*/*z* values) on the surface of dormant JI64 and non-dormant JI92 genotypes (the photo in Figure 4a shows the imaged seed coats). Homogenenous distribution of HLFA over the major parts of seed coats surface of both genotypes was observed and significantly higher signal of HLFA can be seen at the surface of JI64. However, Figure 4d demonstrates that hilum and strophiole (small brown part occurring near hilum, see Figure 4c) contain a significantly lower content of HLFA (dihydroxyoctacosanoate, *m*/*z* 455.4103, in this case, see mass images at Figure 4d). The distribution of the other HLFA exhibits similar pattern as given in supplementary material (Figure S6).

These differences should be related to anatomical structure–presence of chemically different surface layers (e.g., counterpalisades in hilum area) [4]. Our data point to a possible relationship of strophiole and hilum to the water uptake. Those two anatomical parts were already suggested to play a role during imbibition by Karaki et al. [33]. We should remind that no signal of those hydroxylated fatty acids was observed on internal surface (see Figure 3c,d) of dormant/nondormant testa at the same experimental conditions confirming that the changes in HLFA distribution in seed coat are related only to its outermost layers. Consequently, LDI-MSI was used for analysis of HLFA in seed coat cross-sections. These experiments showed that HLFA signals are not present inside of seed coat tissue (Figure 3e,f). The extracellular lipids rich in hydroxylated fatty acids located in the outermost layers of dormant seed coats are probably connected with blocking effect of seed coat to water transport into the endosperm. Hydrophobic effect might be enhanced with structure crosslinking of hydroxyl groups parted on the carbon chain with carboxyl functional group. The distribution of common non-hydroxylated fatty acids over the all seed coat surface is homogenous and their signals are higher in dormant JI64 compared to nondormant JI92. It should be emphasized that the signal of non-hydroxylated fatty acids in strophiole and hilum is not as different from the rest of surface as it is observed in the case of HLFA (supplementary material, Figure S6). This fact could point out the higher significance of HLFA surface distribution with respect to dormancy compared to the distribution of non-hydroxylated FA.

### 2.3. LDI-MS Analysis of HLFA in Recombinant Inbred Lines (RILs)

As previously discussed, our analyses clearly confirmed the significantly higher content of HLFA in seed coat surface of dormant (wild) JI64 genotype compared to non-dormant (domesticated) ones. However, the composition of seed coat (including the fatty acid profile) can be predetermined not only by reproduction regulatory mechanism (physical dormancy) but also by other adaptation traits, i.e., protection from predators, fungi, physicochemical properties of environment. Variance in chemical composition among particular genotypes is expressive as reflected also by visible differences in seed coat morphology. As indicated above, there are certain genotypes relatively similar in morphology but differing strongly in dormancy levels, i.e., JI64 and JI92. Further elimination of differences in another properties and preservation of differences in dormancy at the same time can be achieved genetically by cross of those two genotypes and establishment of recombinant lines (RILs) [5]. The potential of the “RILs approach“ for mapping have been demonstrated by Bagheri et al. which used recombinant inbred line (RIL) population for study of *Brassica rapa* seed metabolites. [34].

**Figure 5:**
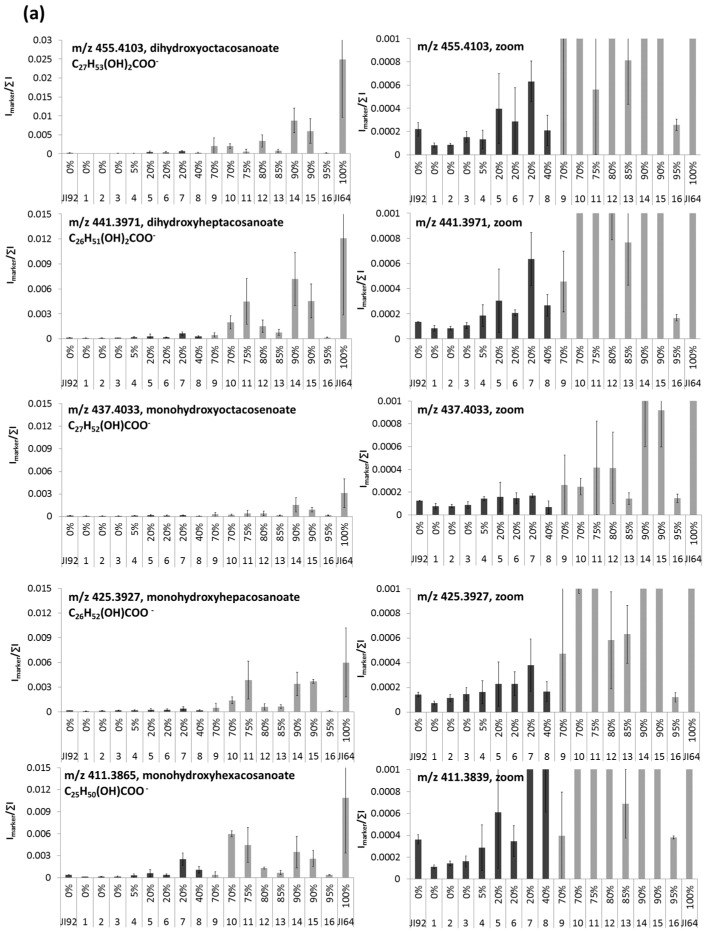

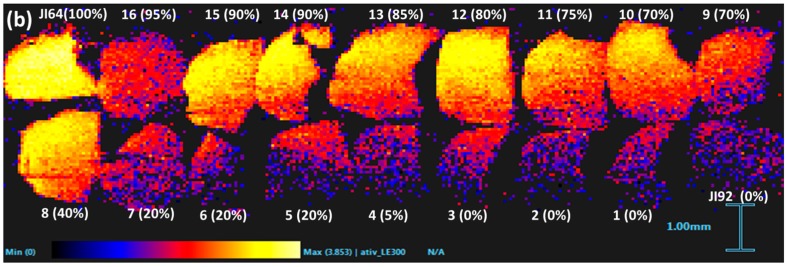
Relationship of HLFA signals to the dormancy level of JI64, JI92 and respective recombinant inbred lines. (**a**) signals of individual hydroxylated long chain fatty acids in parent genotypes and RILs obtained by LDI-MS measurement, black bars denote non-dormant and gray ones dormant lines; signal is expressed as an average of normalized intensities of four repeated measurements; (**b**) distribution of dihydroxyoctacosanoate on the RILs’ surface; numbers at x-axis denote particular RIL lines (1–16), numbers given above the codes of lines in graphs or in parenthesis in mass images, respectively, reflect the dormancy level in percents.

The seeds from the sufficiently genetically homozygous sixth generation of phenotypically evaluated RILs were analyzed by LDI/MS and LDI/MSI methods. Figure 5a shows signal intensities of HLFA on the seed coat surface of chosen RIL pea genotypes placed in order of increasing dormancy percentage.

Generally, the higher dormancy level the higher signal of HLFA is obtained. There are only several exemptions from that rule that deserve a further biological study. Those differences are easily visible from simultaneous mass image (Figure 5b and Figure S7d–h). The distribution of fatty acids over the seed coats of RILs is mostly homogeneous except of strophiole and hilum parts of dormant RILs. Those findings strongly support the significance of HLFA for regulation of water uptake by seed and early stages of germination. Our results are in agreement to the recent work of Chai et al. that studied the differences between *Medicago truncatula* wild (D) and mutant (N) seeds and revealed significant reductions in content of long chain acids (especially 18:2 18–OH at individual monomer level) in non-dormant mutants [35].

Note that also palmitic, stearic and oleic acids exhibit some differences among dormant and non-dormant RILs (Figures S7a–c and S8) although, due to missing hydroxyl groups in their chain, their role in extracellular lipid composition is probably somewhat different (they provide only terminal hydrophobic instead of connecting chains of HLFA).

## 3. Materials and Methods

### 3.1. Chemicals

Methanol (gradient grade), palmitic acid (p.a.), oleic acid (p.a.), 2′,4′,6′-trihydroxyacetophenone monohydrate (THAP, p.a.), 4-aminoquinoline (AQ, p.a.), acetone (for HPLC), acetonitrile (gradient grade) and red phosphorus (p.a., standard for TOF calibration) were purchased from Sigma-Aldrich, St. Louis, MO, USA. Ultrapure water from Milli-Q apparatus (Merck, Kenilworth, NJ, USA) was used for preparation of all solutions.

### 3.2. Plant Material

Seeds of *Pisum species*, namely wild dormant (D) pea *Pisum sativum* subsp. *elatius* (JI64, VIR320, L100) and cultivated non-dormant (N) *Pisum sativum* subsp. *sativum* (JI92, Cameor, Terno) [5] were used. *P. elatius* JI64 originates from Turkey and JI92 is cultivated Afghan landrace (e.g., primitive local type) *P. sativum* (both from John Innes Pisum Collection, Norwich, UK). L100 genotype originates from Israel. Wild *P. elatius* VIR320 is from Vavilov Institute Research of Plant Industry (St. Petersburg, Russia) and was used previously for incompatibility study [36]. Cultivated *P. sativum* cv. Cameor (used for pea genome sequencing) originates from INRA France while Czech cv. Terno is commonly used in cultivation and represents modern variety. Cross of cv. Terno with L100 is being used for establishment of introgression lines [37] while F_6_ (sixth generation) recombinant inbred lines (RILs) derived from JI64 and JI92 cross [38] were used for genetic mapping and transcriptomic analysis [5]. JI64 and JI92 pair is less contrasting in term of visual seed coat appearance [4,5] except of differences in seed coat permeability and dormancy. Plants were cultivated in controlled glasshouse conditions during February–April (2016, 2017) and mature seeds were manually harvested.

Mature pea seeds (*Pisum* sp.) were air dried and stored at laboratory temperature in a dark and dry place until the analysis. The dormancy of particular genotypes and lines was measured by standard method [5]. Briefly, twenty five seeds per line were incubated in Petri dishes (9 cm diameter) over two layers of medium speed qualitative filter papers (Whatman, Maidstone, UK, grade 1) wetted with 3 mL of tap water and incubated in a 25 °C incubator in darkness. Imbibition was scored at 24 h intervals based on changes in seed swelling and germination was determined based on the radicle breaking through seed coat. Non germinated seeds were mechanically scarified after 60 days to determine the percentage of viable seeds.

### 3.3. Sample Preparation for Surface Analysis

Seed coats were mechanically separated from embryos and broken into small pieces. These small pieces (approx. 2 mm in diameter) were fixed on stainless steel MALDI plate with outer surface facing up using a double sided tape (Ulith, Prague, Czech Republic). Six repetitions of each genotype/line were measured. The samples with internal surface oriented up were analyzed for comparison as well. Samples were analyzed directly in dry, intact state (LDI-MS) or after spraying with matrix (MALDI-MS; THAP matrix: 1 mg/mL, dissolved in acetonitrile:water, *v*:*v*, 1:1 for positive ion mode; AQ matrix: 1 mg/mL, dissolved in acetone for negative ion mode). The matrix solution was sprayed using standard SunCollect sprayer (SunChrom GmbH, Friedrichsdorf, Germany) on the seed coat samples in successive six layers.

For study of distribution of analytes on cross-section (vertical distribution), samples of seed coats were dissected from dry seed and saturated with 2% sucrose solution under vacuum. Equal volume of cryo-gel (Cryomatrix Shandon, Thermo Fisher Scientific, Waltham, MA, USA) was added to the sample and shaken overnight to improve subsequent sectioning. Saturated samples were mounted into cryo-gel on the alum chuck, frozen down to −25 °C and cut into 20 µm transversal sections [39]. The prepared slices were put on the normal glass slide. The prepared glass slides were stored at laboratory temperature at dark and dry place until analysis.

### 3.4. Instrumentation

Measurements were done using a high resolution tandem mass spectrometer equipped with a vacuum MALDI ion source, a hybrid QqTOF mass analyzer and an ion mobility cell (Synapt G2-S, Waters, Milford, MA, USA). The MALDI source was equipped with a 350 nm 1 kHz Nd:YAG solid state laser. Mass spectra were collected in positive and negative ionization modes in mass range 50–1200 Da. The effect of laser energy was studied in the range 300–450 and laser step rate in the range 1–30, Hexapole RF amplitude in the range 250–450 V and sample plate voltage in the range 0–25 V. MALDI Extraction voltage was set at 10 V, Hexapole Bias at 10 V and Aperture at 5 V. (MA) LDI-MS analyses were performed for 6 min (300 scans were collected). Acetone solution of red phosphorus was used for TOF calibration (supernatant of suspension prepared at concentration 1 mg/mL). The control of the mass spectrometer and data collection were performed using MassLynx 4.1 software (Waters, Milford, MA, USA). Trap collision energy (TrapCE) was set to 4 eV and transfer collision energy to 2 eV for MS scan. For MS/MS experiments the effect of collision energy in both collision cells on the yield of fragments was tested in the range 10–50 eV (the optimal values for particular experiments are given in Results and Discussion). The exact mass measurement involved external calibration and automatic lock mass measurement (correction) using red phosphorus (see above). Spectra for lock mass correction were collected as a part of each analysis prior to seed sample measurement. The identification of selected markers (signals differing significantly between dormant and non-dormant samples) was based on exact mass measurement and consequent target MS/MS experiments.

#### Laser Desorption/Ionization Imaging Mass Spectrometry (LDI-MSI)

LDI-MSI technique was used for analysis of surfaces as well as cross sections of seed coats. HDImaging software (version 1.4, Waters) was used for the setup of experiment and the data collection. The LDI-MSI measurement of seed coat surface was taken in negative ionization mode with 60 µm laser beam size. Four laser energies (200, 250, 300, 350) were tested in order to find the highest yield of signals of studied markers (details are given in Results and Discussion). Laser repetition rate was set at 1000 Hz and scan time at 1 s. The Hexapole RF Amplitude was the same as given in Results and Discussion. Filtration of low abundant signals was applied during measurement to decrease the number of datapoints (the number of the most intense peaks included in raw datafile was set to 300,000 and the signal threshold was set to 10 counts). No matrix was used for MSI experiments.

### 3.5. Data Processing

The raw MS data were firstly processed by MarkerLynx XS software that is an extension of MassLynx platform (Waters). This software provided extraction, normalization and alignment of data (creation of data matrix). Parameters of optimized MarkerLynx XS method were set as follows: marker intensity threshold 1000 (optimal value for resolution of dormant and non-dormant species in PCA plot), peak separation 0.05 Da, mass range 50–1200 Da. “Combined Scan Range” was used as the type of analysis. The obtained data matrices were studied by multivariate statistics. Principal Component Analysis (PCA) and Orthogonal Projections to Latent Structures Discriminant Analysis (OPLS-DA) were applied using EZinfo software (version 2.0, Umetrics, Malmö, Sweden) with Univariate or Pareto scaling. Significance of obtained markers was evaluated according to their position in appropriate S-plots.

## 4. Conclusions

LDI-MS in combination with PCA and OPLS-DA proved to be a useful tool for the classification (distinction) of dormant and non-dormant genotypes of pea by the direct analysis of seed coat outer surface. Minimum sample treatment is necessary, even the application of matrix can be omitted and seeds can be analyzed in dry (inactive) state. Particular dormant genotypes exhibited strong differences in signals of dormancy markers suggesting differences in the process of their water imbibition. Detailed study of morphologically similar but in dormancy levels strongly different pair of genotypes, i.e., JI64 and JI92 and derived RILs, revealed significant differences in content of hydroxylated long chain fatty acids (ranging from C26 to C28). Significantly higher signals of HLFA were found in dormant JI64 and dormant RIL lines.

The effect of experimental conditions on the signal of HLFA was studied in detail. Application of matrix caused mechanical changes of samples and matrix free analysis provided better results. Hexapole RF amplitude, sample plate voltage, laser energy and laser step rate appeared to be significant LDI parameters for HLFA signal and their values were optimized. Ion mobility experiments allow effective filtration of HLFA signals from ballast ones. Laser desorption/ionization mass imaging experiments reveal homogeneous distribution of HLFA on the outer seed coat surface with the exemption of strophiole and hilum in JI64 seed coat that show significantly lower content of HLFA. Analysis of JI64–JI92 recombinant inbred lines strongly supports the relation of physical dormancy with the distribution of HLFA. The obtained information contributes to a deeper insight into the mechanism of water absorption by seed that is important also for food and agricultural research.

## Figures and Tables

**Table 1:** List of fatty acids found among dormant markers by LDI-MS, MALDI-MS and OPLS-DA analysis. (* deviation of measured mass (*m*/*z*) from theoretical value calculated from elemental composition).

Elemental Composition	Matrix Free Experiments, Figure 3a				Matrix Assisted Experiments, Figure 3b			
	[M–H]^−^	dtm (ppm) *	Coordinates in S-Plot		[M–H]^−^	dtm (ppm) *	Coordinates in S-Plot	
			CoeffCS(2)	p(corr)(1)			CoeffCS(2)	p(corr)(1)
C_16_H_31_O_2_^−^	255.2331	0.78	0.00125	0.90637	255.2326	−1.18	−0.00012	−0.09994
C_18_H_33_O_2_^−^	281.2393	−33.07	0.00049	0.74660	281.2530	15.64	0.00017	0.70174
C_18_H_35_O_2_^−^	283.2613	−10.24	0.00066	0.85055	283.2625	−6.00	0.00002	0.04306
C_26_H_51_O_3_^−^	411.3850	2.67	0.00162	0.80646	411.3816	−5.59	0.00079	0.92125
C_27_H_53_O_3_^−^	425.3990	−1.18	0.00107	0.81814	425.3963	−7.52	0.00065	0.96154
C_28_H_53_O_3_^−^	437.3946	−11.20	0.00067	0.77322	437.3929	−15.09	0.00042	0.91775
C_27_H_53_O_4_^−^	441.3950	1.36	0.00160	0.83272	441.3978	7.70	0.00087	0.96626
C_28_H_55_O_4_^−^	455.4065	−7.69	0.00249	0.84469	455.4138	8.34	0.00126	0.91329

## Supplementary Materials

Supplementary materials can be found at www.mdpi.com/1422-0067/18/10/2196/s1.
