# Addendum: Cechová, M. et al. Towards Better Understanding of Pea Seed Dormancy Using Laser Desorption/Ionization Mass Spectrometry. *Int. J. Mol. Sci.* 2017, *18*, 2196

**DOI:** 10.3390/ijms18122771

**Published:** 2017-12-20

**Authors:** Monika Cechová, Markéta Válková, Iveta Hradilová, Anna Janská, Aleš Soukup, Petr Smýkal, Petr Bednář

**Affiliations:** 1 Regional Centre of Advanced Technologies and Materials, Department of Analytical Chemistry, Faculty of Science, Palacký University, 17. Listopadu 12, 771 46 Olomouc, Czech Republic; monicechova@gmail.com (M.C.); ponizilova.marketa@seznam.cz (M.V.); 2 Department of Botany, Faculty of Science, Palacký University, Šlechtitelů 27, 783 71 Olomouc, Czech Republic; HradilovaI@seznam.cz (I.H.); petr.smykal@upol.cz (P.S.); 3 Department of Experimental Plant Biology, Faculty of Science, Charles University, Viničná 5, 128 44 Prague, Czech Republic; janska@natur.cuni.cz (A.J.); asoukup@natur.cuni.cz (A.S.)

It has been brought to our attention that one funding project of Ministry of Education, Youth and Sports of the Czech Republic (LO1417) was missing in the Acknowledgement section of our published paper [1], and therefore we would like to add it and report the Acknowledgements as follows:

**Acknowledgments:** The support of the Czech Science Foundation (14-11782S and 16-21053S), Ministry of Education, Youth and Sports of the Czech Republic (LO1305 and LO1417), and Palacký University Olomouc (IGA_PrF_2017_020) is gratefully acknowledged.

This addendum does not cause any changes to the results and conclusions in the original published paper.
