# Supplementary material for: Towards Better Understanding of Pea Seed Dormancy Using Laser Desorption/Ionization Mass Spectrometry
Source: Int J Mol Sci. 2017 Oct 21;18(10):2196. doi: 10.3390/ijms18102196 (PMC5666877; doi:10.3390/ijms18102196)
Supplement: Supplementary file 1 [file ijms-18-02196-s001.zip › Supplement - Tables EXCEL/ijms-230718 suppl. Figures.docx]

# Supplementary Material


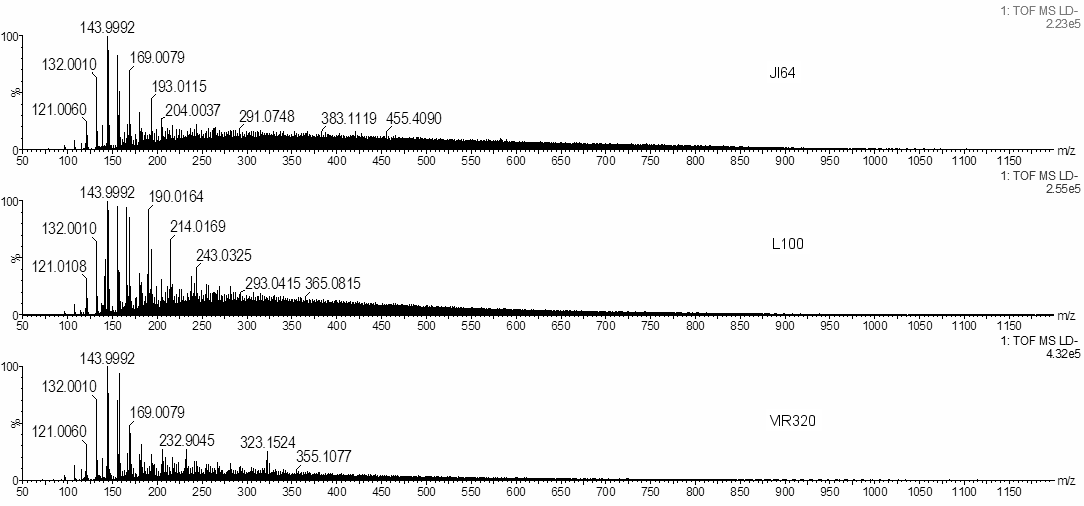

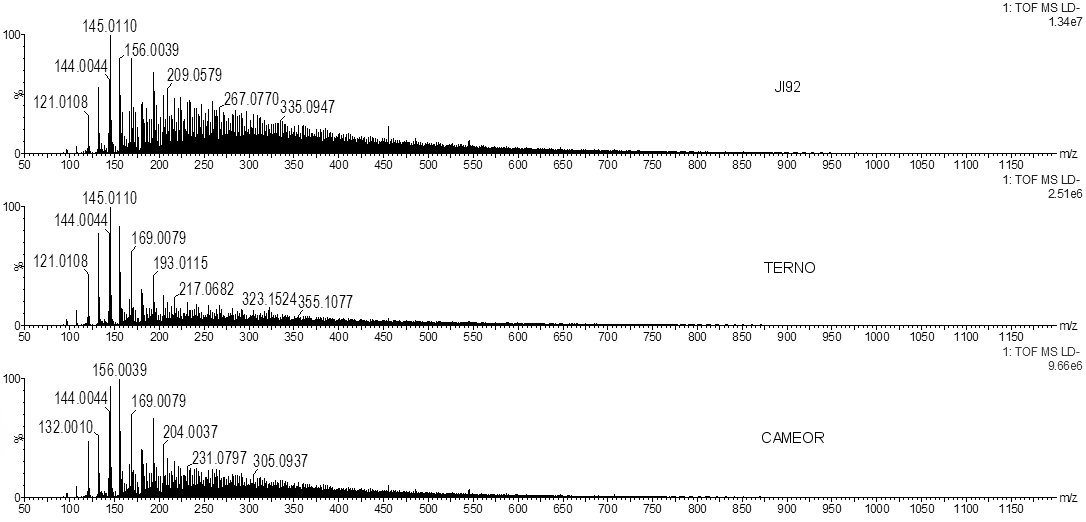


**Figure S1.** (**a**)–LDI-MS spectra of six genotypes measured in negative ionization mode. **(**dormant—JI64, L100, VIR320; nondormant –JI92, TERNO, CAMEOR)


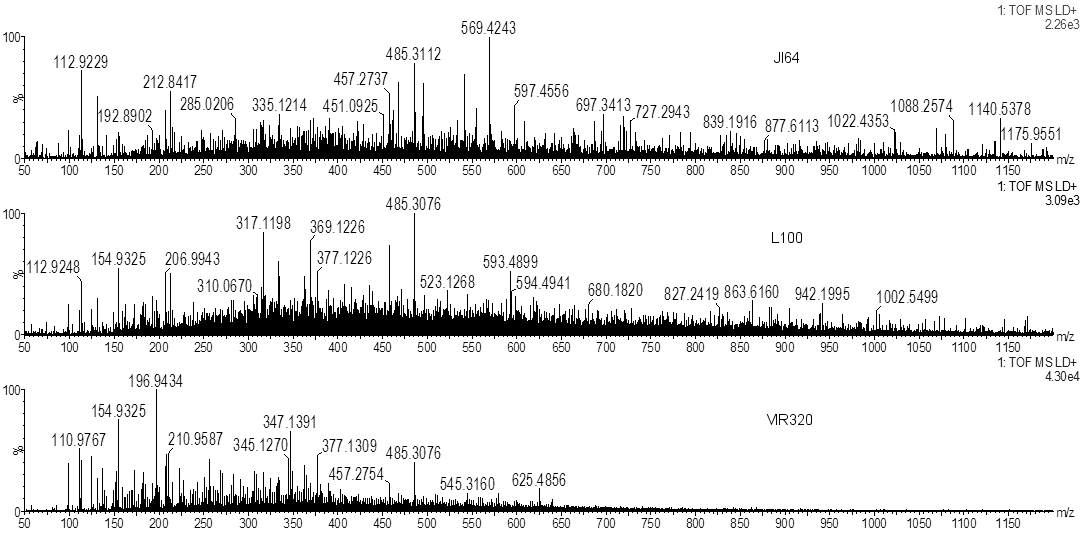

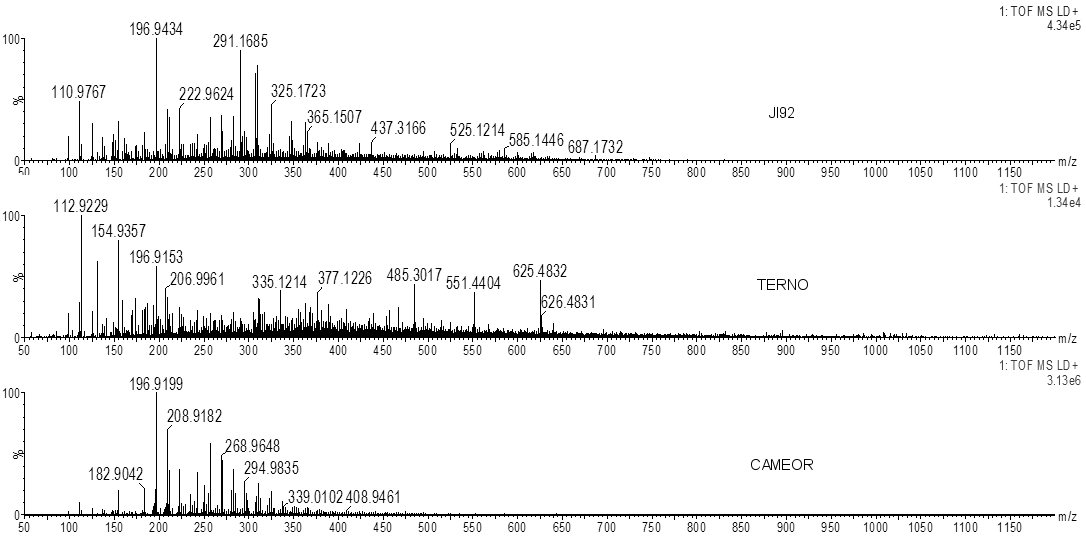


**Figure S1.** (**b**)—LDI-MS spectra of six genotypes measured in positive ionization mode. (dormant—JI64, L100, VIR320; nondormant –JI92, TERNO, CAMEOR)


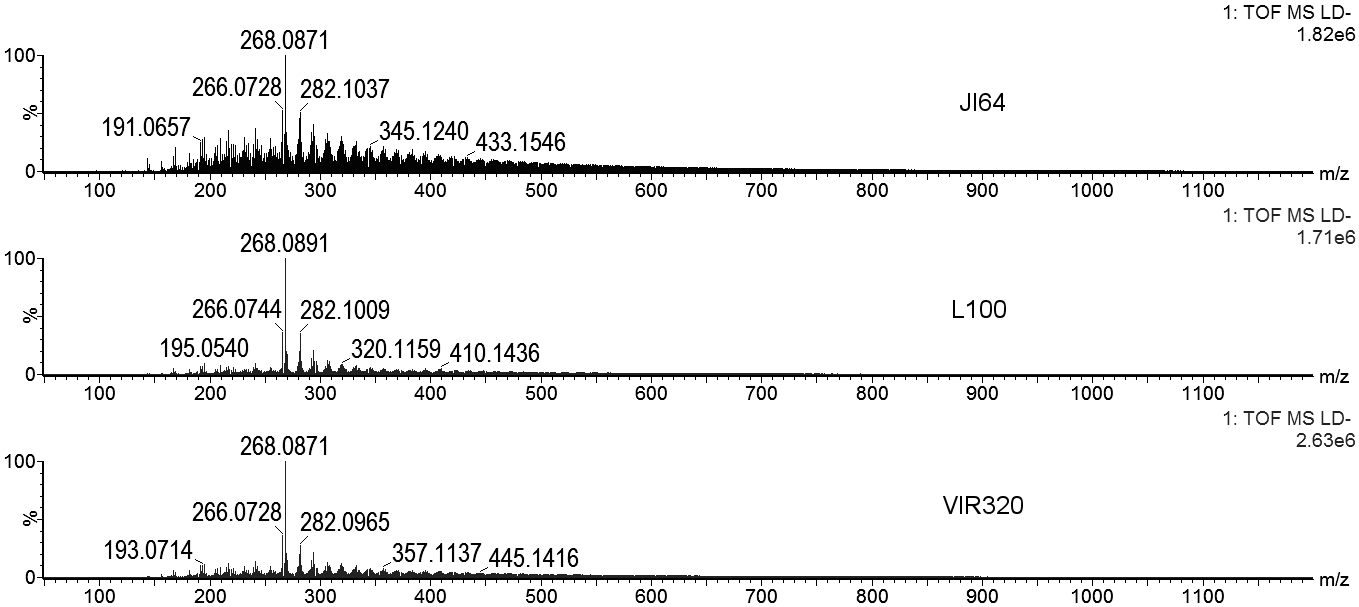


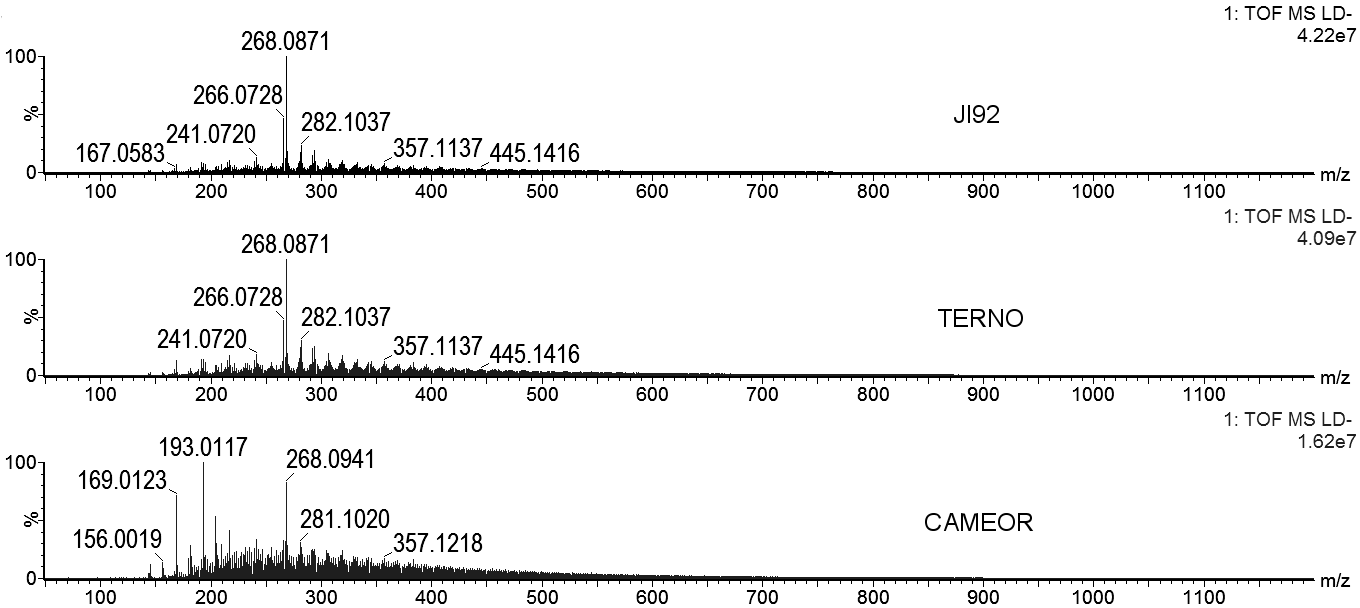


**Figure S1.** (**c**)—MALDI-MS spectra of six genotypes measured in negative ionization mode. (dormant—JI64, L100, VIR320; nondormant –JI92, TERNO, CAMEOR)


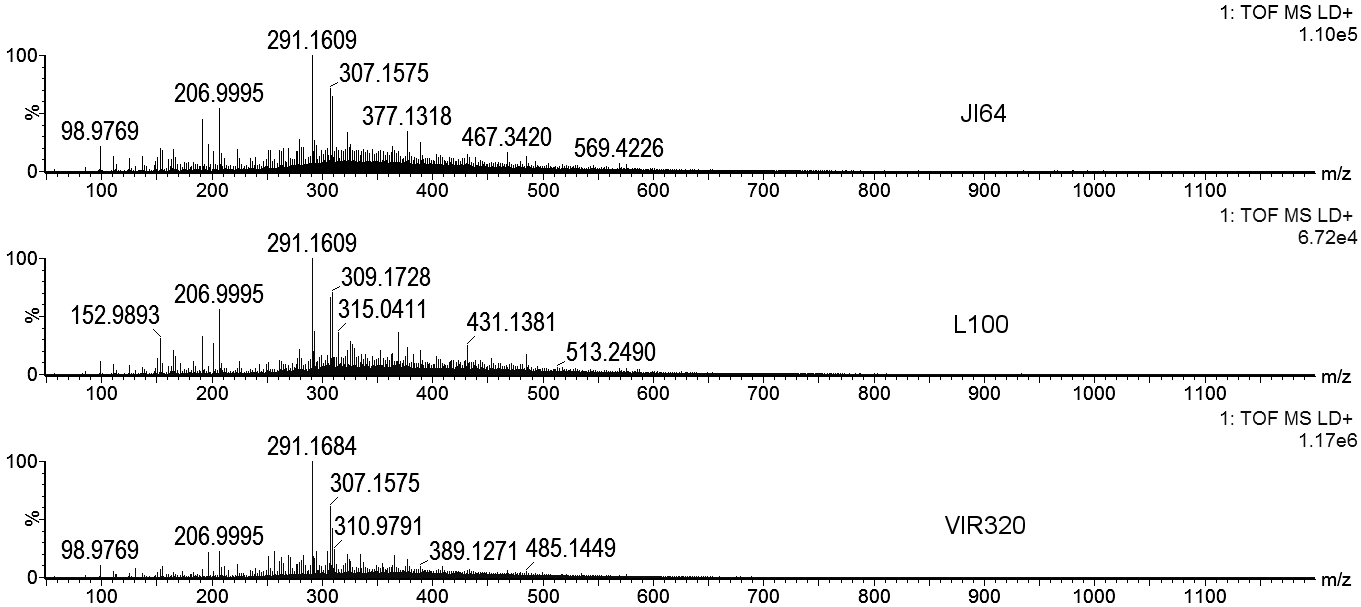


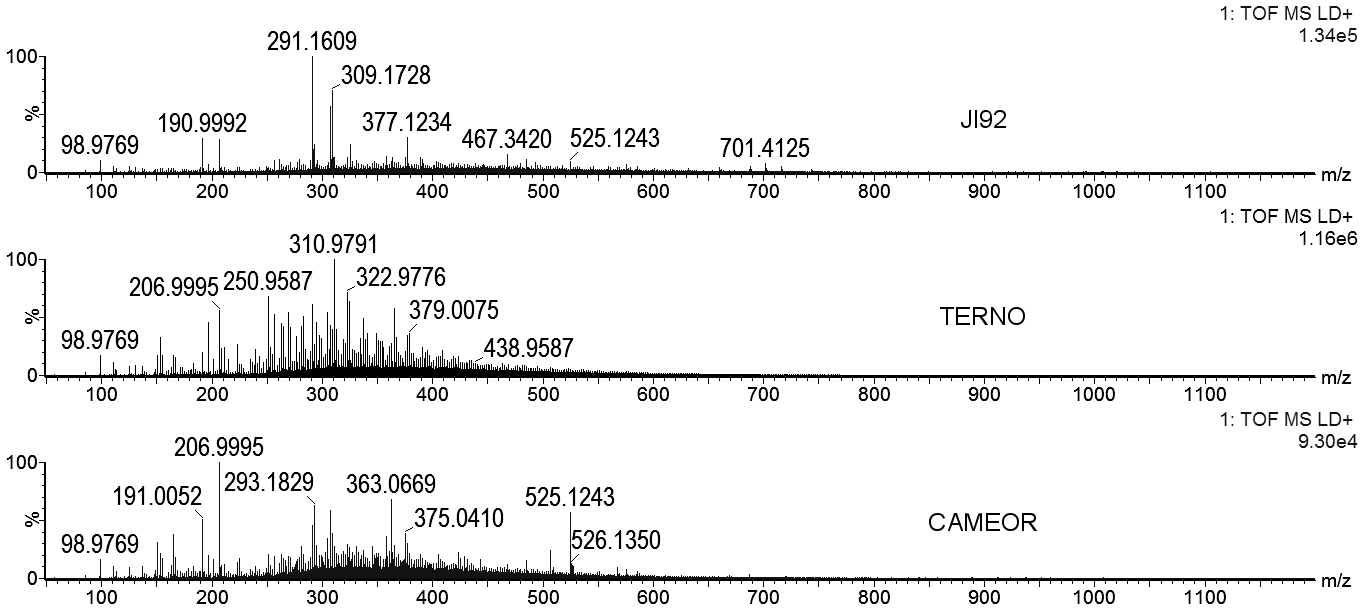


**Figure S1.** (**d**)—MALDI-MS spectra of six genotypes measured in positive ionization mode. (dormant—JI64, L100, VIR320; nondormant –JI92, TERNO, CAMEOR).


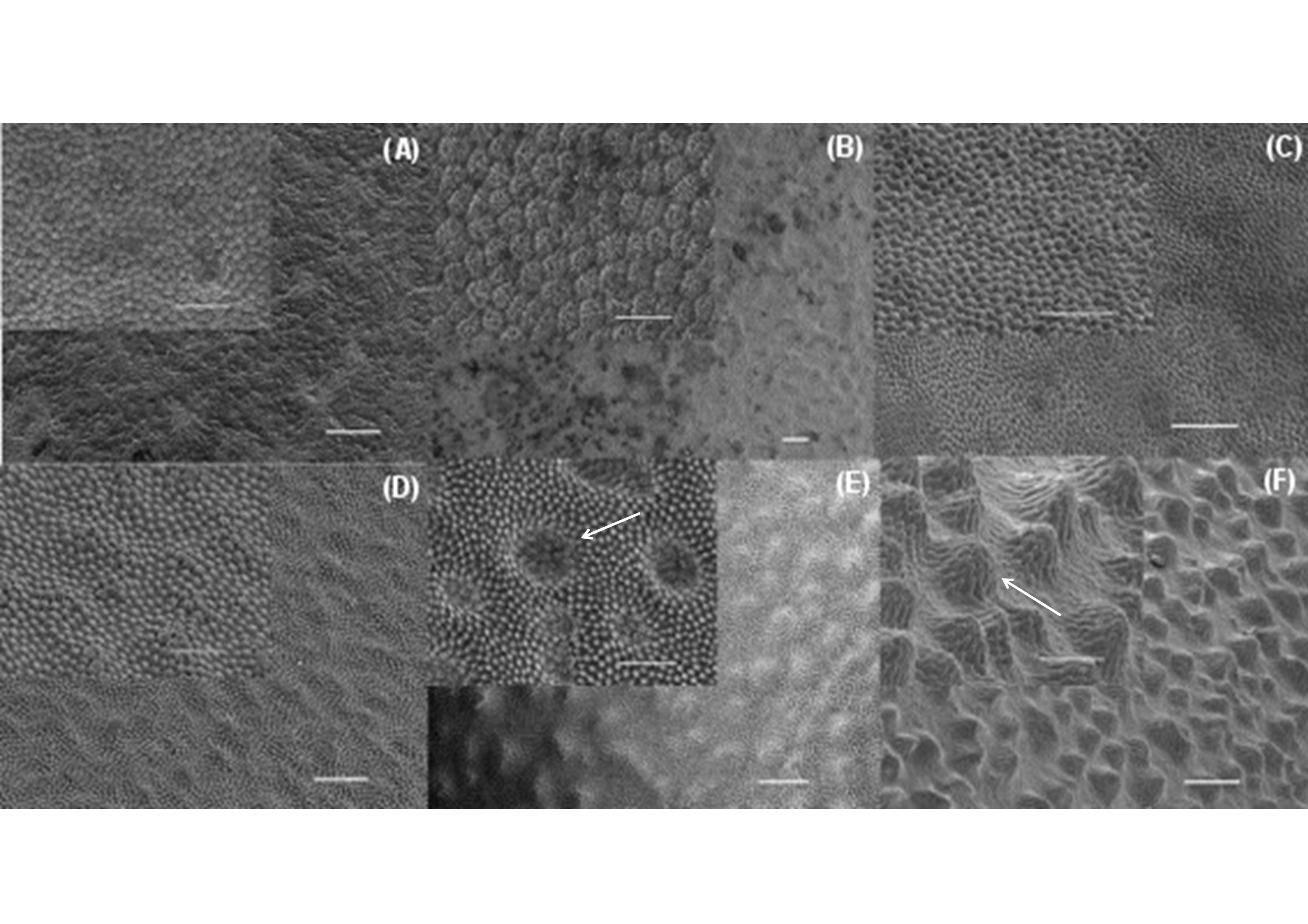


**Figure S2.** Scanning electron microscopy of outer seed coat surface
(**a**)—CAMEOR, (**b**)—TERNO, (**c**)—JI92, (**d**)—VIR320, (**e**)—L100, (**f**)—JI64; overall views, scale bars =100 µm; details, scale bars = 50 µm. SEM show smooth surface of four pea seed coats (CAMEOR, TERNO, JI92 and VIR320) whereas seed coat surface of L100 and JI64 is covered with “gritty“ structures (see white arrows).


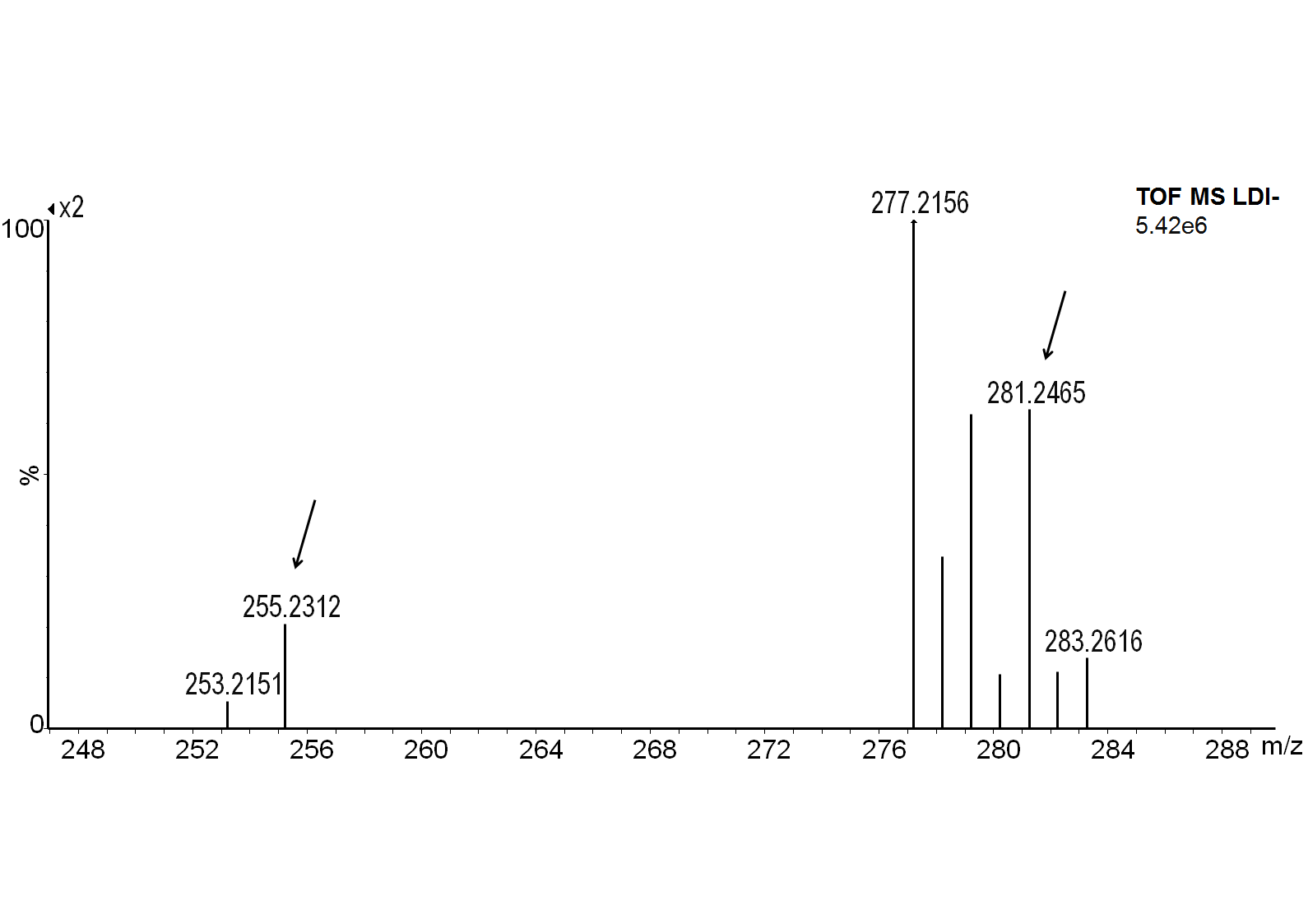


**Figure S3.** (**a**)—LDI-MS spectrum of standards of palmitic and oleic acids in negative ionization mode.


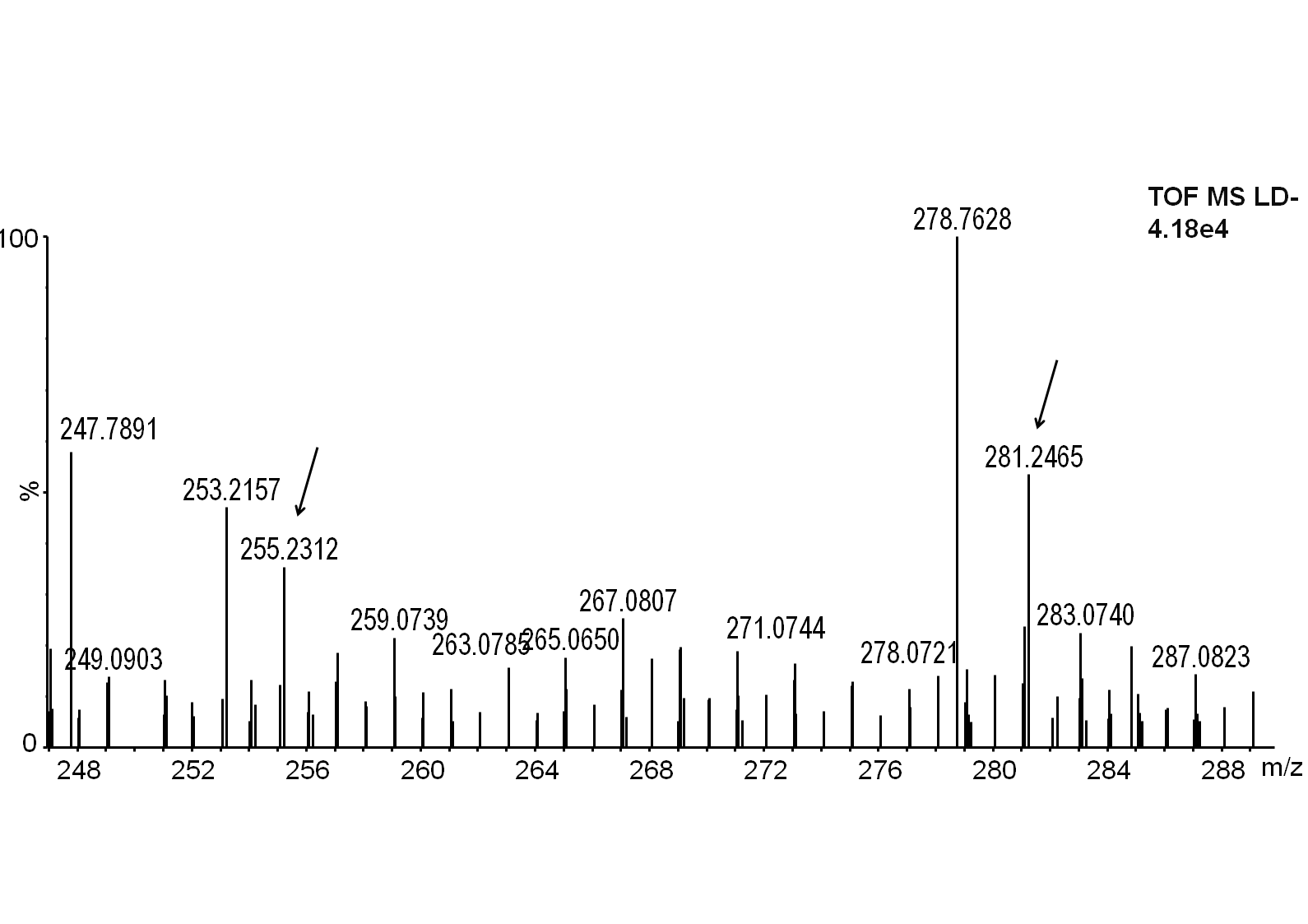


**Figure S3.** (**b**)—LDI-MS spectrum of JI 64 seed coat in negative ionization mode.


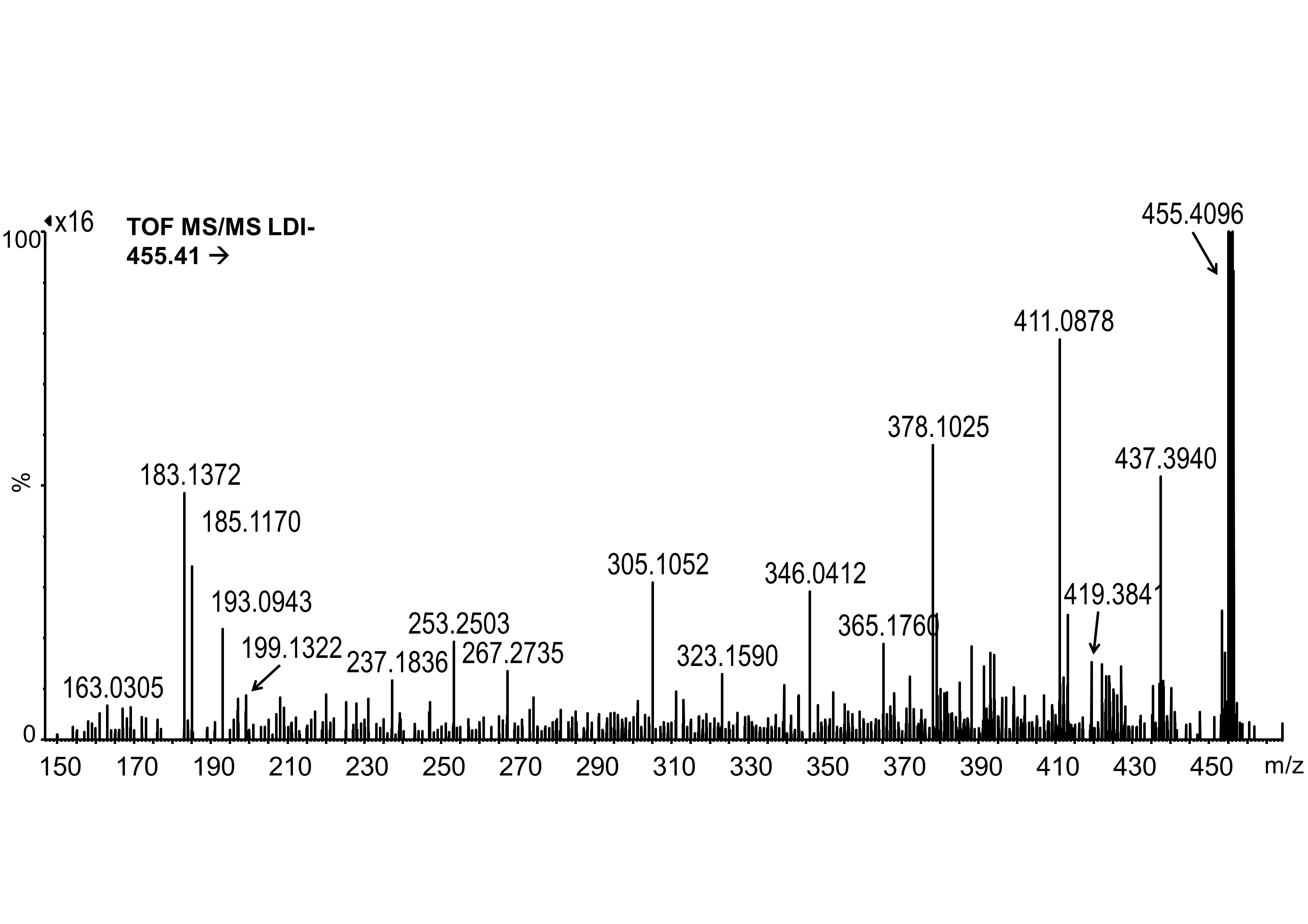


**Figure S4.** MS/MS spectrum of dihydroxyoctacosanoate in negative ionization mode.


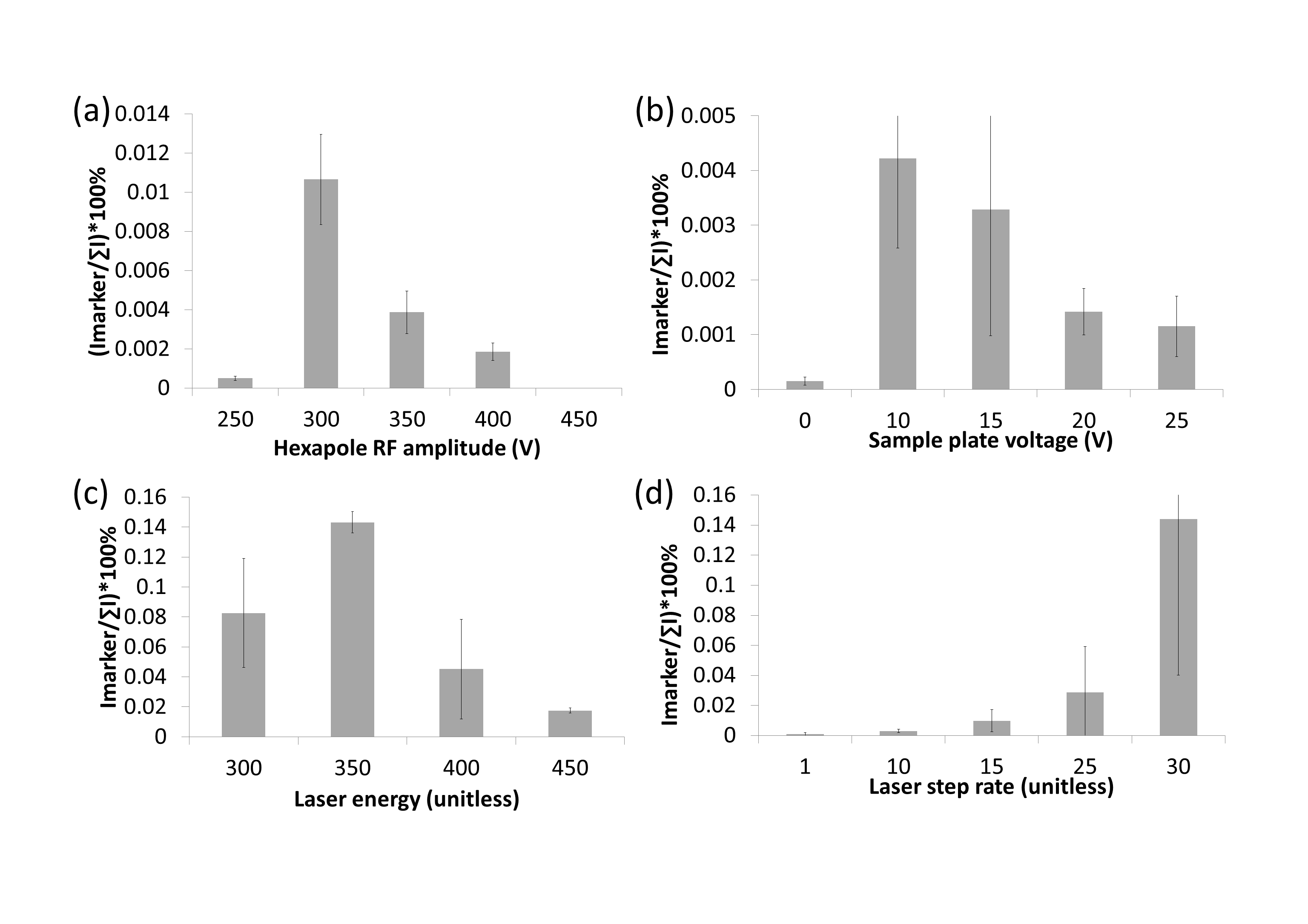


**Figure S5.** Optimization of LDI source parameters for dihydroxyoctacosanoate signal. (**a**)**—**effect of hexapole RF amplitude of dihydroxyoctacosanoate signal, (**b)—**effect of sample plate voltage**,** (**c**)**—**effect of laser energy, (**d**)**—**effect of laser step rate.


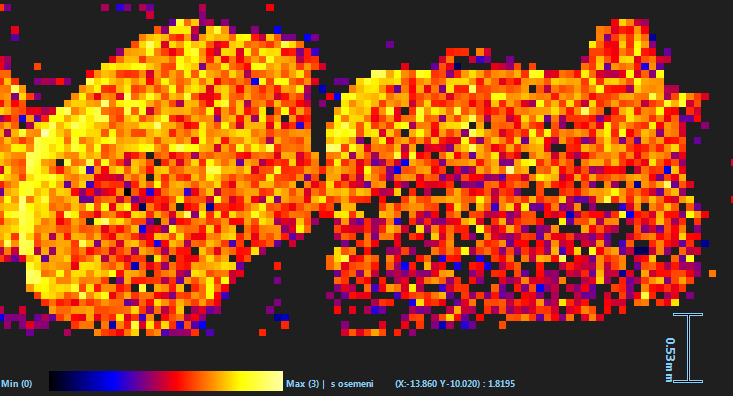

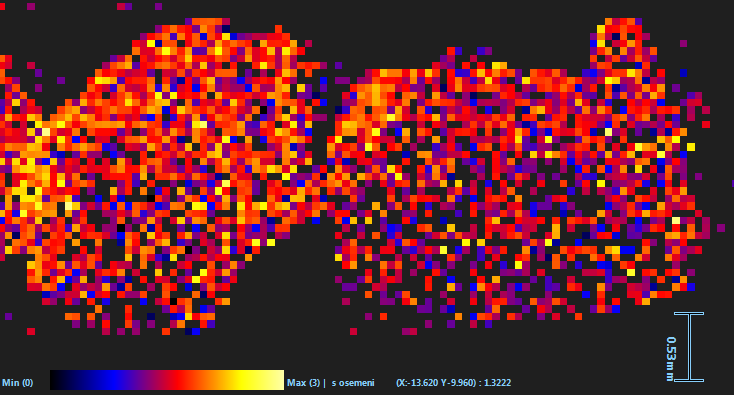

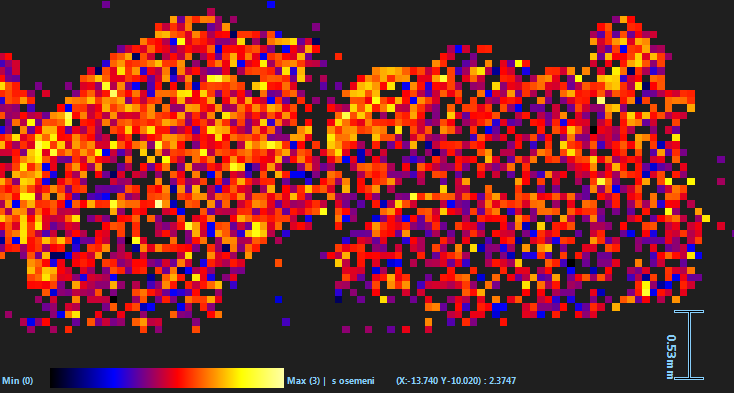

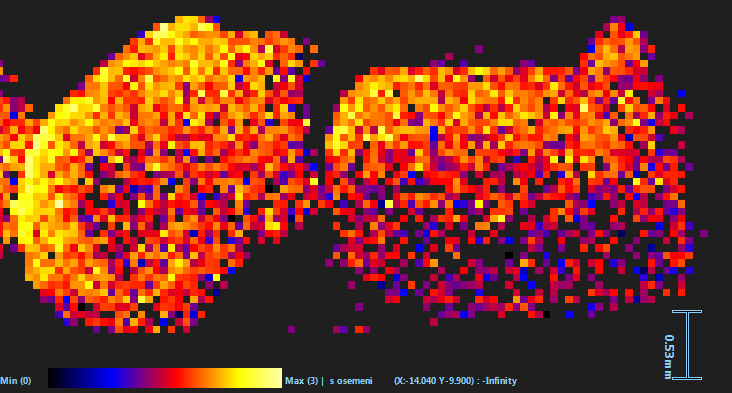

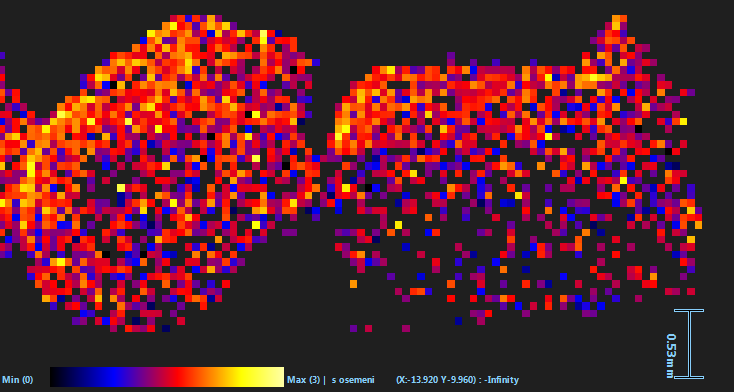

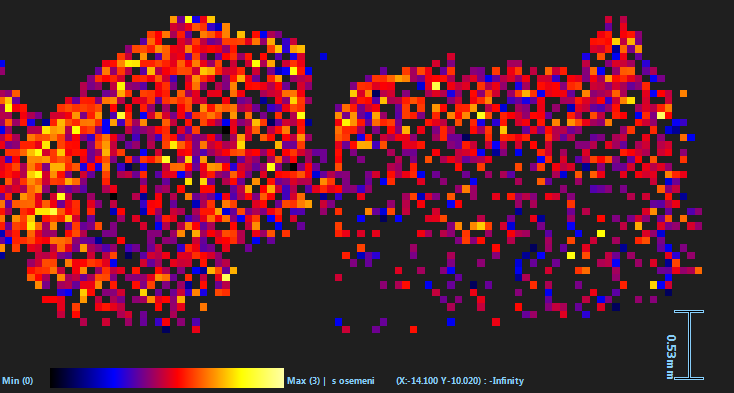

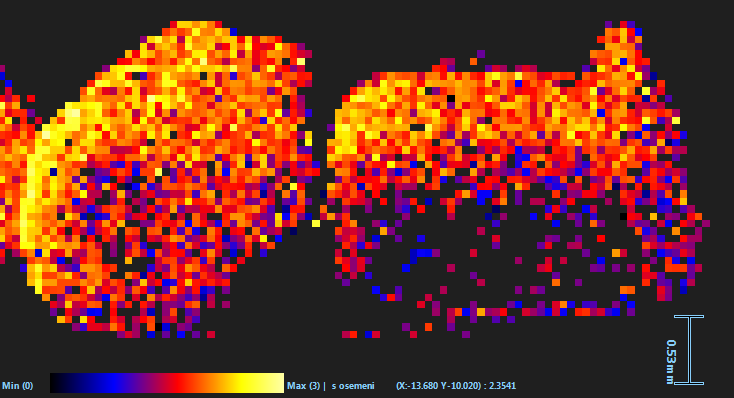

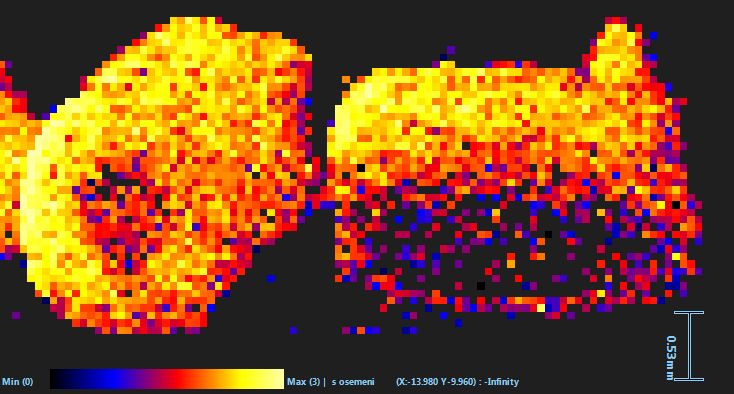


(h))

(g))

(f))

(e))

(d))

(c)

(b)

(a)

**Figure S6.** Surface distribution of dormancy markers on the external surface of JI64 genotype seed coats (**a**)—palmitate, (**b**)—oleate, (**c**) – stearate, (**d**)—Hydroxyhexacosanoate, (**e**)—hydroxyheptacosanoate, (**f**)—hydroxyoctacosenoate, (**g**)—dihydroxyheptacosanoate, (**h**)—dihydroxyoctacosanoate.


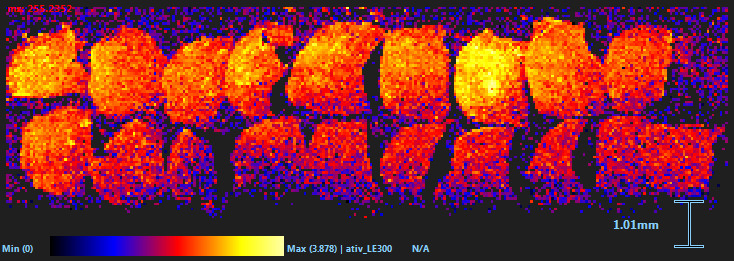

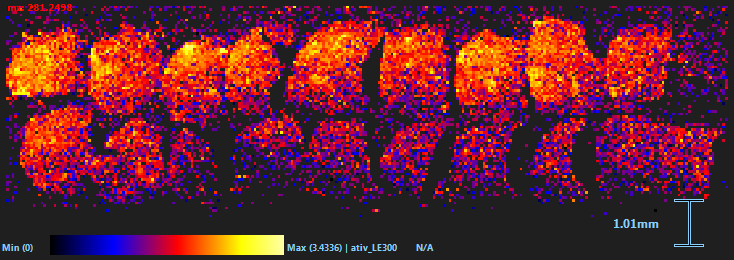

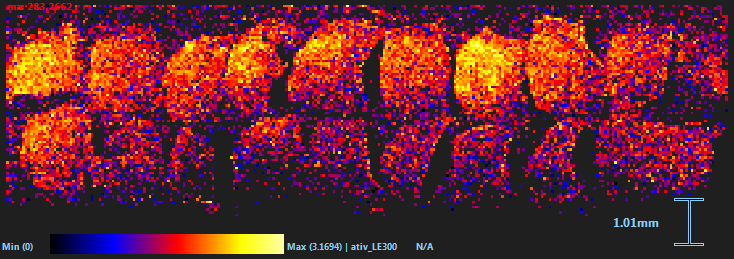

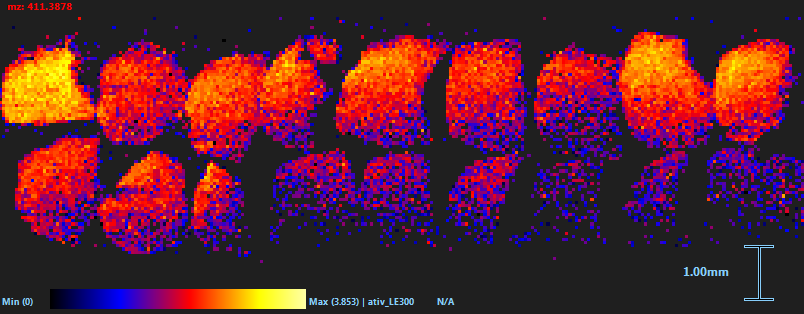

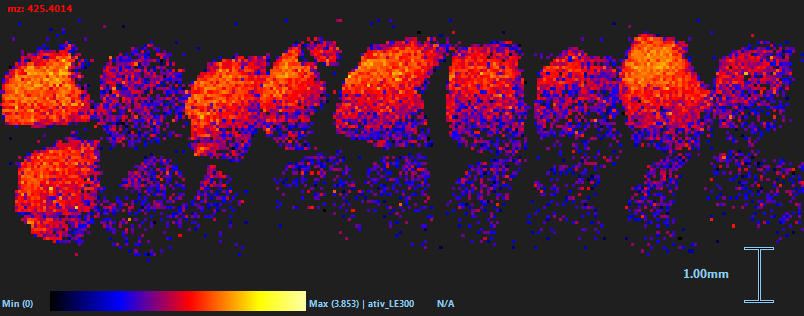

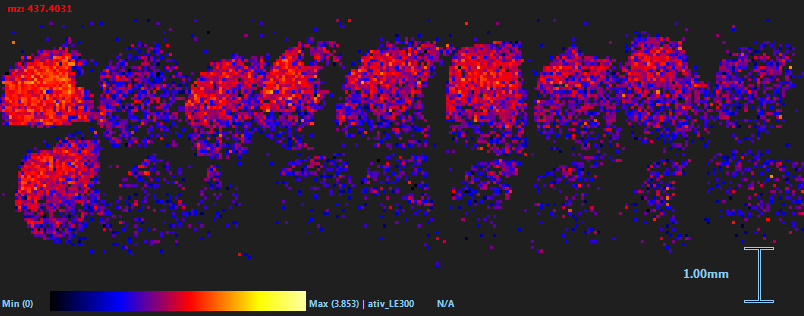

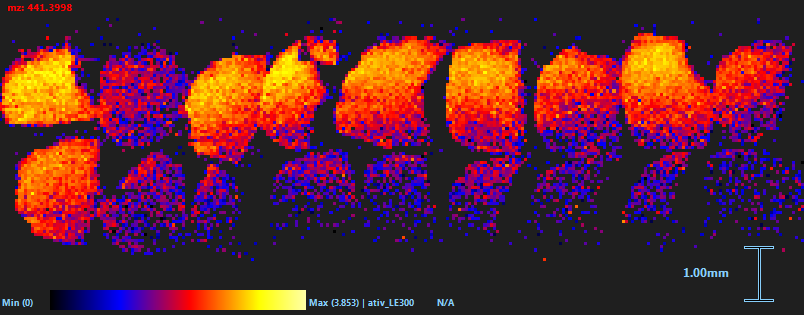

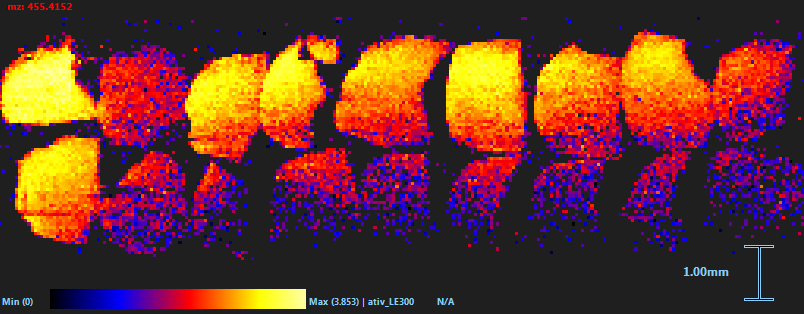


(e)

(h)

(g)

(f)

(d)

(c)

(b)

(a)

**Figure S7.** Surface distribution of dormancy markers on the external surface of recombinant inbred lines (JI64xJI92, F6) seed coats: (**a**)—palmitate, (**b**) —oleate, (**c**) —stearate, (**d**) —hydroxyhexacosanoate, (**e**) —hydroxyheptacosanoate, (**f**) —hydroxyoctacosenoate, (**g**) —dihydroxyheptacosanoate, (**h**) —dihydroxyoctacosanoate/


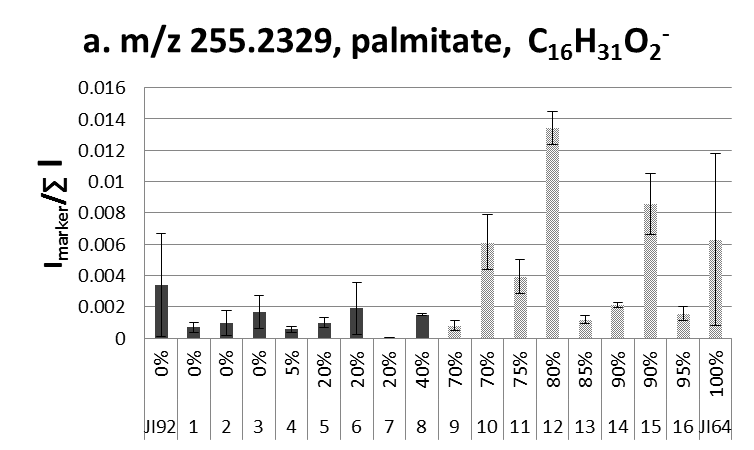

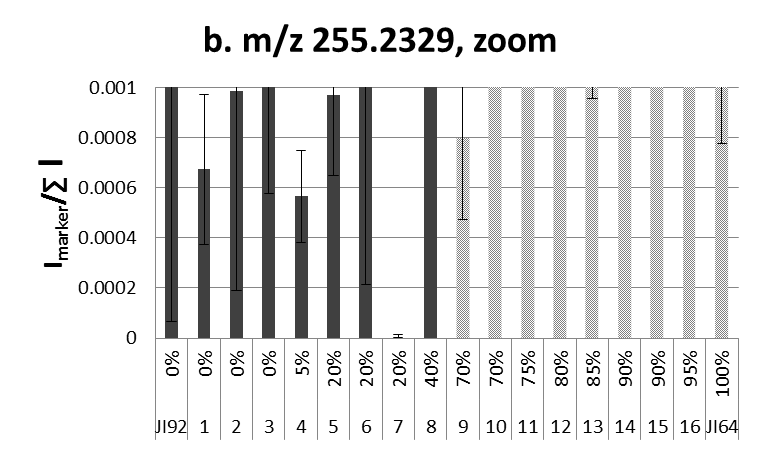


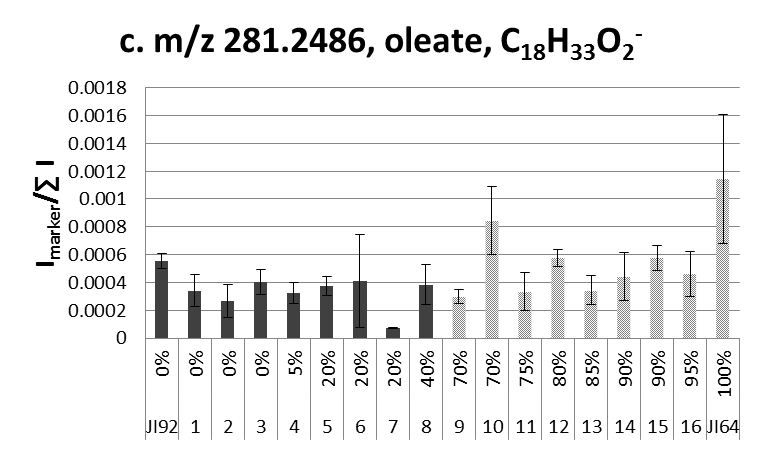

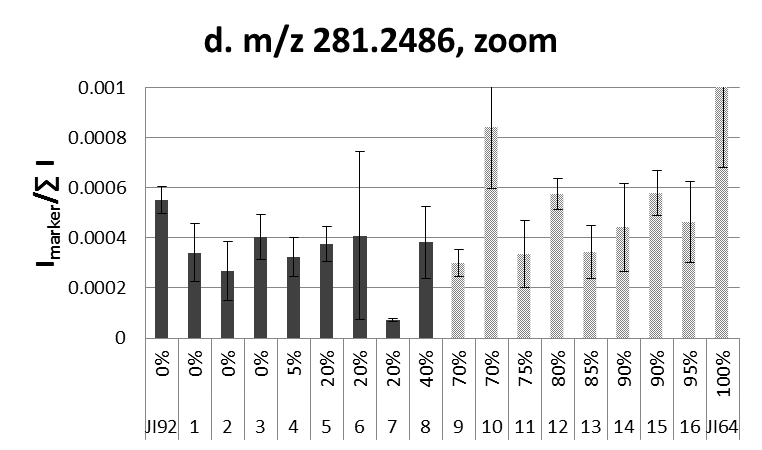


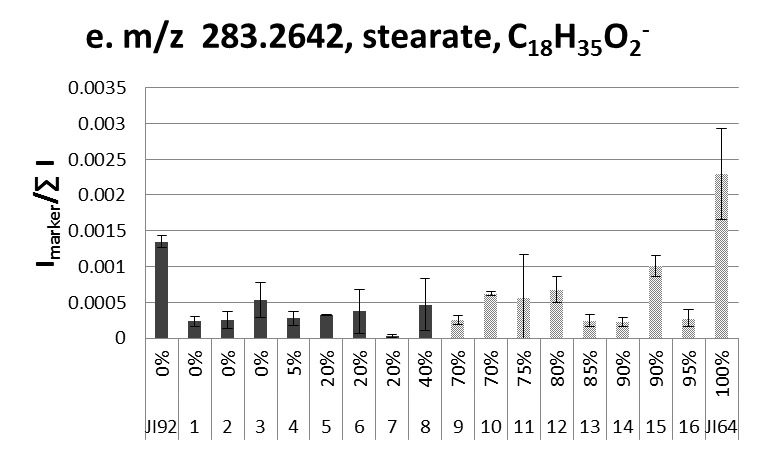

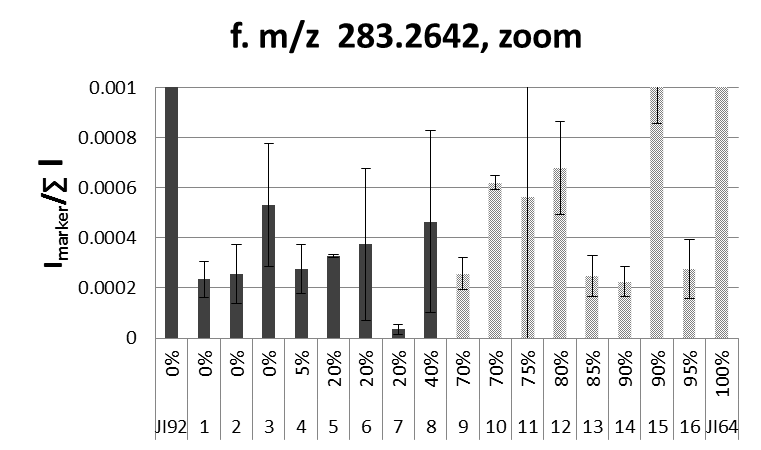


**Figure S8.** Comparison of the LDI-MS signals of common fatty acids in recombinant inbred lines (JI64 x JI92, F6). Signal is expressed as average of normalized intensities.

**Table S2.** The most significant dormancy markers observed for particular dormant genotypes against all non-dormant genotypes found by OPLS-DA. (the significance of the obtained markers was expressed in order of product value of their coordinates in particular S-plots; the markers expressed consistently in two dormant genotypes are given in italics)

| **Order** | **Genotype JI64** | | | **Genotype L100** | | | **Genotype VIR320** | | |
| --- | --- | --- | --- | --- | --- | --- | --- | --- | --- |
|  | ***m/z*** | **Coordinates in S-plot** | | ***m/z*** | **Coordinates in S-plot** | | ***m/z*** | **Coordinates in S-plot** | |
|  |  | **CoeffCS** | **p(corr)** |  | **CoeffCS** | **p(corr)** |  | **CoeffCS** | **p(corr)** |
| 1 | **455.4066** | 0.002264 | 0.7473 | **190.0180** | 0.004405 | 0.7720 | **323.1417** | 0.003473 | 0.7995 |
| 2 | **441.3945** | 0.001403 | 0.7270 | **166.0208** | 0.004344 | 0.7821 | **232.9015** | 0.003596 | 0.7544 |
| 3 | **411.3821** | 0.001384 | 0.6981 | ***214.0227*** | 0.003448 | 0.7740 | **321.135** | 0.002437 | 0.8353 |
| 4 | **402.6586** | 0.001337 | 0.6784 | **142.0196** | 0.003066 | 0.7997 | ***205.9496*** | 0.003220 | 0.5954 |
| 5 | **456.4127** | 0.001190 | 0.7379 | **165.0404** | 0.003262 | 0.7505 | **169.9492** | 0.003360 | 0.5608 |
| 6 | **243.0345** | 0.001307 | 0.6456 | **141.0337** | 0.002247 | 0.7396 | **144.0012** | 0.003291 | 0.5443 |
| 7 | **439.4140** | 0.001084 | 0.7665 | **238.0219** | 0.002316 | 0.6786 | **256.8998** | 0.002583 | 0.6818 |
| 8 | **247.7957** | 0.001187 | 0.6663 | **158.0143** | 0.002039 | 0.6273 | **181.9495** | 0.002701 | 0.5928 |
| 9 | **186.9772** | 0.000966 | 0.7910 | **240.0293** | 0.001524 | 0.8119 | **267.1969** | 0.002025 | 0.7323 |
| 10 | **309.7387** | 0.001076 | 0.6799 | **157.9491** | 0.001640 | 0.6010 | **239.2003** | 0.001773 | 0.8356 |
| 11 | **430.0949** | 0.000826 | 0.7911 | **243.0345** | 0.001638 | 0.5984 | **325.1689** | 0.002041 | 0.6850 |
| 12 | **267.0338** | 0.001032 | 0.6233 | **262.0542** | 0.001329 | 0.7275 | **138.9738** | 0.002360 | 0.5733 |
| 13 | **477.0936** | 0.000750 | 0.8304 | **242.0199** | 0.001397 | 0.6294 | **307.1682** | 0.001500 | 0.6768 |
| 14 | **418.0929** | 0.000777 | 0.8006 | **241.0161** | 0.001465 | 0.5897 | **233.9025** | 0.001177 | 0.8624 |
| 15 | **425.3914** | 0.000888 | 0.6988 | **138.0232** | 0.001179 | 0.7091 | **280.8994** | 0.001606 | 0.6208 |
| 16 | **438.0930** | 0.000757 | 0.8184 | **395.3849** | 0.001484 | 0.5634 | **305.154** | 0.001789 | 0.5518 |
| 17 | **439.0973** | 0.000755 | 0.8121 | **314.0325** | 0.001311 | 0.6159 | **131.9998** | 0.001769 | 0.5485 |
| 18 | ***214.0227*** | 0.000888 | 0.6637 | **290.0280** | 0.001353 | 0.5941 | **223.1711** | 0.000980 | 0.9149 |
| 19 | **453.0921** | 0.000738 | 0.7905 | ***205.9496*** | 0.001147 | 0.6819 | **237.1907** | 0.000975 | 0.8895 |
| 20 | **494.1131** | 0.000676 | 0.8397 | **255.0275** | 0.001351 | 0.5551 | **195.1393** | 0.000958 | 0.9052 |
